# Association Between Pulse Wave Velocity and Cerebral Small Vessel Disease: A Scoping Review

**DOI:** 10.7759/cureus.96142

**Published:** 2025-11-05

**Authors:** Zaneh Kahook, Oren Nedjar, Amanda Escudero, Caitlin Montgomery, Jamie Ropelewski, Harvey N Mayrovitz

**Affiliations:** 1 Osteopathic Medical School, Nova Southeastern University Dr. Kiran C. Patel College of Osteopathic Medicine, Fort Lauderdale, USA; 2 Medical Education, Nova Southeastern University Dr. Kiran C. Patel College of Allopathic Medicine, Davie, USA

**Keywords:** arterial stiffness, brachial-ankle, cardiovascular risk, carotid-femoral, dementia, stroke, vascular biomarkers, vascular disease

## Abstract

Cerebral small vessel disease (CSVD) is a common condition affecting the small vessels of the brain and is often linked to dementia and strokes. Its prevalence increases with age, making noninvasive predictors essential for early intervention and management. Arterial stiffness, commonly measured via pulse wave velocity (PWV), has been proposed as a marker of CSVD, but differences in PWV measurement methods, CSVD markers, and confounders such as age and vascular risk complicate this relationship. This scoping review aimed to clarify the association between PWV and CSVD by searching four databases (EMBASE, Ovid MEDLINE, Web of Science, and CINAHL) for peer-reviewed articles written in English published between 2014 and 2024 that included both PWV and at least one major CSVD biomarker: (1) white matter hyperintensities (WMH), (2) lacunar infarcts (LI), (3) fractional anisotropy (FA), (4) cerebral microbleeds (CMB), or (5) perivascular spaces (PVS). Of 1,345 articles initially identified, 53 met the inclusion criteria. The findings suggest a general trend of higher PWV being associated with increased WMH volume, greater PVS severity, decreased FA, and the presence of LI, with several studies also linking the CSVD-PWV association to cognitive decline, stroke recurrence, and depression. The most definitive associations were found between PWV and the presence of WMH. These findings support PWV’s potential as an early predictor of CSVD severity and progression, provided that suitable standardization of measurement methods and further longitudinal research are done. The association between PWV and cognitive decline, stroke risk, and functional recovery further underscores PWV in a potential role as a predictive marker in CSVD management. Identifying PWV as a modifiable risk factor emphasizes the importance of early detection, lifestyle intervention, and cardiovascular risk management in mitigating CSVD progression and outcomes.

## Introduction and background

Cerebrovascular disease (CVD) is a group of etiologically diverse disorders that may lead to brain parenchymal damage [1] and, depending on severity, may lead to cognitive decline [2,3]. Cerebral small vessel disease (CSVD) is the most common CVD [4] and is the most common vascular cause of dementia [2]. CSVD has been reported to be associated with some common vascular disorders, including dyslipidemia, hypertension, and diabetes [1,2,5]. The prevalence of CSVD increases with age, rising from around 5% in people aged 50 to nearly 100% by age 90, and contributes to 45% of dementia cases and 25% of strokes [6]. Strokes are the first leading cause of disability and the fifth leading cause of death in the United States, making it a significant public health concern [7].

Although the pathology of CSVD is not fully understood, some hypothesize that it is due to endothelial damage of the brain’s blood vessels and exposure of the brain parenchyma to reactive oxygen and nitrogen species [1]. Multiple potential effects of CSVD include demyelination, diffuse axonal injury, and neuronal apoptosis [8], which occur as a result of ischemia from CSVD pathology [1,3,8]. On MRI imaging, CSVD can present as small subcortical infarcts, white matter hyperintensity (WMH), lacunae, microbleeds, and perivascular spaces (PVS) [2].

It has been suggested that increased carotid artery stiffness is an independent risk factor for CVD [9]. Furthermore, it has been reported that aortic stiffness is independently associated with cognitive decline, particularly affecting memory, processing speed, and executive function [10]. A question of interest concerning CSVD is its potential linkage or relationship to readily measured noninvasive cardiovascular parameters. One such parameter is arterial stiffness, which is noninvasively determined by arterial pulse wave velocity (PWV) measurements. PWV is the speed at which cardiac-generated pressure waves travel through the arteries [11] and is considered to be the "gold standard" for measuring arterial stiffness [12]. PWV is measured by determining the transit time of the pulse wave between two separated vascular locations, with a greater PWV implying a stiffer arterial system [1,2,13-15], and is often used as a surrogate indicator of arterial health [16]. Various methods are used to measure PWV, differing in the locations of the proximal and distal sites, with the carotid-femoral artery PWV (cfPWV) being one and the brachial-ankle PWV (baPWV) another [17-19]. 

Some linkages between increased PWV and the potential effects of CSVD have been described in the literature. These include a greater volume of white matter lesions, particularly in individuals with uncontrolled hypertension [20], low cerebral blood perfusion [21], and as a marker of increased cognitive decline [22]. However, one study reported that while PWV strongly correlates with both age and cerebrovascular changes, its values fail to accurately reflect true vascular health because of not including the beneficial effects of lifelong exercise [23]. Furthermore, since multiple methods of assessing PWV have been employed [13], considering the possible impacts of such measurement variability is useful. 

Although there are prior reviews that report some aspects of associations between PWV and markers of CSVD, there are some limitations related to the inclusion of relevant studies in which both PWV and CSVD indicators were considered [24,25]. In these otherwise excellent reviews, there are qualified restrictions on the type of cerebral assessment used, the type of measurement method used, and the features of the patients included. Moreover, conclusions drawn can conflict, as different methods of measuring PWV may lead to varying results [2]. Studies may also use different markers of cerebrovascular disease or do not consider alternative measures for measuring cognitive impairment [24,25]. 

Based on these considerations, it appears that a detailed mapping of the association between PWV and CSVD, considering variability of measurement methods, age aspects, and other potential confounding features, may be useful to help further characterize the extent of such a relationship. Hence, this review aims to address this gap by systematically examining available evidence on the relationship between arterial PWV and CSVD to clarify its potential clinical relevance and diagnostic value.

## Review

Search strategy

**Figure 1:**
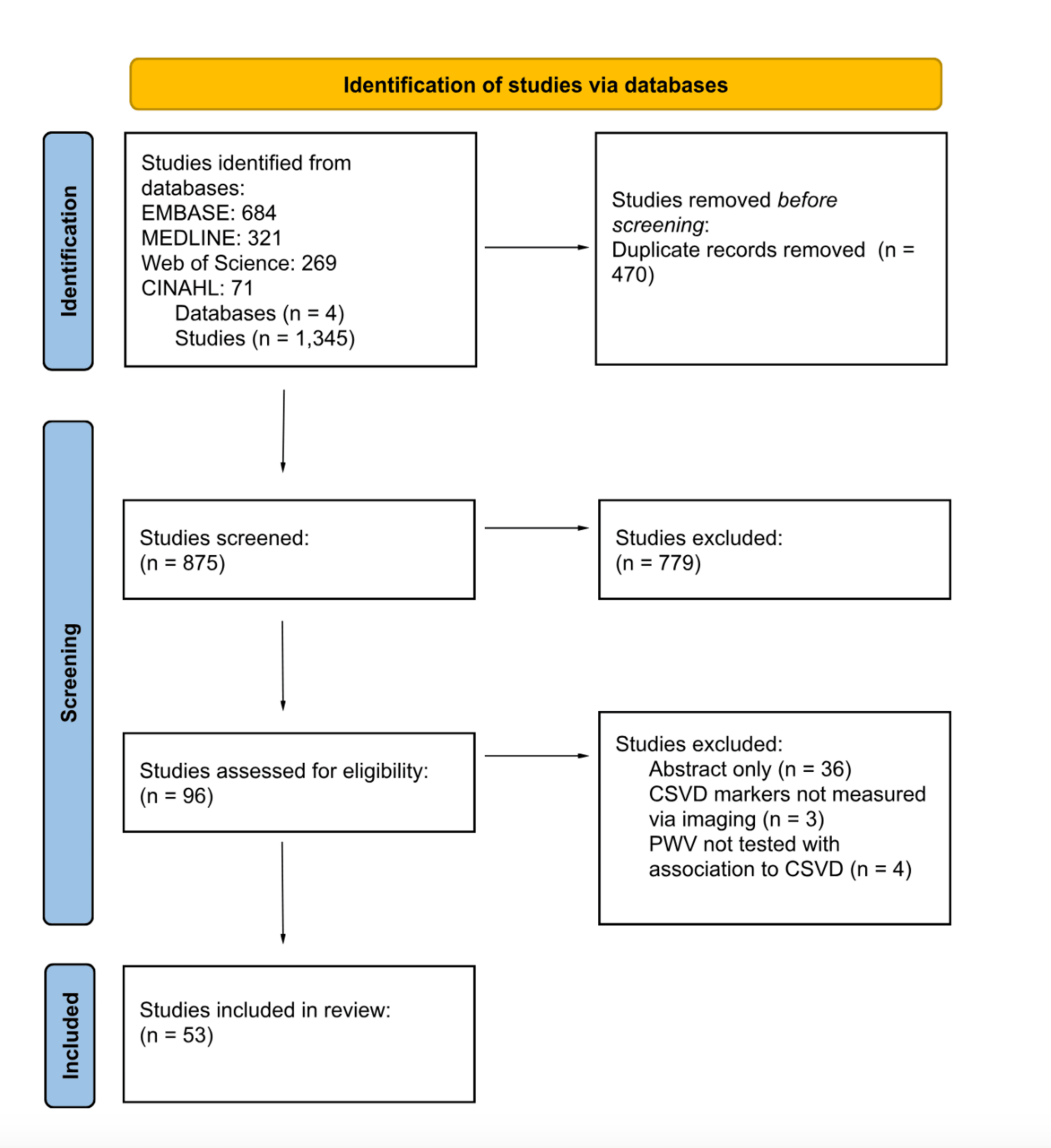
The Preferred Reporting Items for Systematic Reviews and Meta-Analyses (PRISMA) flowchart

A search strategy was built by analyzing key terms, and searches were carried out using Excerpta Medica Database (EMBASE), Ovid Medical Literature Analysis and Retrieval System Online (MEDLINE), Web of Science, and Cumulative Index to Nursing and Allied Health Literature (CINAHL). Data collection took place in September 2024 using the same search process for each database. The search terms "pulse wave velocity" and "cerebral small vessel diseases" were entered into the controlled descriptors for each database. The Boolean operator AND was used for simultaneous occurrences, and OR for their synonyms. The search strategy was peer-reviewed by a librarian using the Peer Review of Electronic Search Strategies (PRESS) checklist. The details of the search terms and the whole search strategy can be found in the Appendices. Briefly, to be included in this review, articles needed to be peer reviewed, written in English, and published between 2014 and 2024, with patients 18 years old and diagnosed with CSVD. Diagnosis of CSVD was based on MRI or CT findings. To ensure that the review captured the diverse manifestations of the umbrella term CSVD, markers considered were WMH, lacunar infarcts (LI), PVS, cerebral microbleeds (CMB), and fractional anisotropy (FA). Excluded articles, if present, were other scoping reviews, systematic reviews, case reports, animal research, and reports with abstracts only. The Preferred Reporting Items for Systematic Reviews and Meta-Analyses (PRISMA) flowchart method was utilized to streamline the inclusion process of the studies (Figure 1).

Selection of sources of evidence

The search identified 1,345 citations, as outlined in Figure 1. First, 470 duplicates were removed using the Rayyan automatic software (Rayyan Systems Inc., Cambridge, MA, USA), leaving 875 studies to be screened. Two authors read the titles and abstracts of the first 438 articles, while two other authors reviewed the other 437 articles. Each author independently conducted the abstract review process, included articles solely from the 2014-2024 range, and agreed on which articles were relevant for further consideration (n = 96 articles). The full text of these 96 articles was obtained, and two authors independently read each and followed the pre-established inclusion criteria to guide their decisions for each article. The reviewers then discussed each full-text article that was being considered for inclusion. If there were disagreements among the reviewers, the two reviewers presented the article to a third independent reviewer. Discussions ensued between all three reviewers until an agreement occurred. The final number of studies included was 53.

The quality and validity of each article were central to the selection process. The 53 included studies underwent a rigorous screening using the Joanna Briggs Institute Critical Appraisal Tools to assess the risk of bias. All 53 articles met the criteria and were retained for the final scoping review. This comprehensive appraisal supported an in-depth discussion of each study’s relevance and quality within the research context.

Rayyan and Excel (Microsoft Corp., Redmond, WA, USA) managed and organized the data. These tools helped track participant information, study context and concepts, research methods, and key findings relevant to the review [26]. Data collected in Rayyan and Excel were then transferred into the PRISMA diagram, which was completed according to the established inclusion and exclusion criteria.

Total CSVD burden findings

A higher PWV (cfPWV or baPWV) was found to be significantly associated with a higher total CSVD burden, using WMH, LI, CMB, and PVS as markers in the CSVD burden score [22,27-29]. CSVD burden refers to the overall extent of brain damage caused by small vessel disease, typically reflected in markers such as WMH, LI, CMB, and PVS. Moreover, each CSVD marker was often but not always found to be independently associated with a higher PWV. In one study, LI showed no significant association with PWV, while in another, deep CMB, but not strictly lobar, were significantly associated [27,29]. Interestingly, it was found that higher baPWV was associated with severe PVS in white matter, larger WMH volume, and lower brain parenchymal fraction (indicating brain atrophy), but was only slightly associated with strictly lobar CMB [30]. baPWV was also determined to be associated with acute and chronic CSVD, and optimal baPWV cutoff values were identified as 13.12 m/s for CSVD presence and 15.63 m/s for severe CSVD [22,29]. However, a more recent study reported no link between baPWV and CSVD severity in acute stroke patients after controlling for confounding factors, especially age [31]. 

In a cohort study with patients who had a recent small subcortical infarct, higher 24-h PWV was associated with a higher CSVD score, and baseline CSVD burden was the main determinant of new microbleeds and/or vascular lacunae after a follow-up period of two years [32]. In stroke-free individuals, those with a higher baPWV had a significantly higher prevalence of CSVD, irrespective of their blood pressure status [33]. Moreover, among participants not using antihypertensive agents, baPWV remained an independent risk factor for CSVD, while blood pressure did not [33]. It was further found that higher cfPWV was associated with lower cognitive performance, and this was partly mediated by microvascular dysfunction score, which included CSVD markers WMH, LI, CMB, and retinal vessel dilation [10]. However, carotid stiffness showed no significant association with cognitive dysfunction. These findings were reinforced by a study reporting that higher aortic PWV was associated with CSVD markers (WMH, LI, CMB, and PVS) and cognitive impairment, especially among those with cardiovascular disease [34]. CSVD mediated the relationship between hypertensive exposure and cognitive impairment, suggesting CSVD is a pathway linking hypertension with cognitive decline. In patients with type 1 diabetes mellitus (T1DM), those with CSVD (LI, CMB, WMH) had a higher central PWV (cPWV) compared to those without any signs of CSVD [35]. However, this was not independent of arterial risk factors (systolic blood pressure, BMI, LDL, etc.) in T1DM, and baPWV did not show any difference between those with and without CSVD. Patients with T1DM also had a higher prevalence of CSVD, reinforcing the increased vascular burden in this population. Overall, these findings highlight an association between higher PWV and increased CSVD burden, with evidence linking PWV to individual CSVD markers, cognitive impairment, and vascular risk.

White matter hyperintensities 

Subjects with higher PWV, whether measured through aortic PWV, cfPWV, or baPWV, were significantly associated with increased WNH volume [36-39]. The association between cfPWV and WMH, as well as the association between baPWV and WMH, remained consistent across individuals aged 45 years and older [30,40,41]. Aortic PWV was directly associated with the progression of WMH, suggesting that WMH advancement can be tracked through PWV measurements, with higher PWV indicating a greater increase in WMH volume [36]. However, a few studies have reported null or attenuated associations between PWV and CSVD markers after adjusting for age and cardiovascular risk factors [31,42-44]. In hypertensive male patients, higher cfPWV was significantly associated with greater WMH volume, lower cerebral blood flow, and increased risk of intracranial stenotic plaques [45]. Interestingly, one study found that the coronary artery calcification (CAC) score mediated the association between baPWV and WMH in males [46]. Furthermore, higher cfPWV was associated with increased WMH volume, reduced total brain and Alzheimer’s disease signature region volumes, and poorer performance in executive function and general cognition [47]. This association with cognition was further supported by findings linking higher cfPWV to poorer processing speed, larger lateral ventricular volumes, and a greater burden of WMH [40]. Higher baseline aortic PWV was associated with greater reductions in gray matter volume in the hippocampus and occipital lobe and greater increases in WMH volume in the temporal lobe over time [48]. Moreover, it was observed that a higher cfPWV was associated with more depressive symptoms, and this relationship was mediated in part by WMH volume and subcortical infarcts [49]. In subjects with type 2 diabetes mellitus (T2DM), there was a greater increase in WMH volume than in subjects without T2DM [50]. Overall, these findings underscore the intricate links between PWV, WMH progression, depression, and cognitive decline, highlighting the impact of vascular health and related risk factors.

Fractional anisotropy and brain microstructure changes

One study found that increased cfPWV was associated with lower FA and higher mean diffusivity (MD) across the brain, indicating reduced white matter microstructural integrity [51]. These associations were stronger in participants with higher WMH volumes. cfPWV remained a significant predictor of lower white matter integrity in this study, even after adjusting for WMH volume and other covariates. In contrast, one study reported no association between cfPWV and FA in middle-aged adults, and another found a negative association between cfPWV and FA in asymptomatic middle-aged men [52,53]. However, both articles reported a positive association between cfPWV and increased white matter free-water content [52,53]. Moreover, higher cfPWV was associated with increased retinal vessel widening, a proxy for cerebral microvascular change [54]. These findings highlight the complex relationship between cfPWV, white matter integrity, and brain microstructure, suggesting a link between increased pulse wave velocity and greater microvascular brain injury.

Lacunar infarcts 

Several studies found a significant relationship between higher baPWV and the presence of LI. One article reported a strong correlation between baPWV and LI, establishing baPWV as a reliable marker for detecting CSVD [55]. Similarly, an article identified that higher baPWV was significantly associated with the presence of silent lacunar infarcts [37], and another observed that a higher cardio-ankle vascular index (CAVI) was significantly associated with infarct expansion in LI patients [56].

In patient populations with specific cardiovascular risk factors, aortic PWV, baPWV, and cfPWV also demonstrated associations with lacunar infarcts [57-59]. In T2DM patients, higher aortic PWV was significantly associated with LI [57]. Likewise, in coronary artery disease (CAD) patients without prior stroke, higher baPWV was significantly associated with the presence of intracranial lesions (ICL), including LI, with baPWV acting as an independent predictor [58]. Additionally, patients with ICL had worse cardiovascular outcomes, with a higher incidence of adverse events over the follow-up period. It was reported that in the presence of lacunae, higher cfPWV was associated with a greater CSVD, with stronger associations observed in participants with poorly controlled hypertension [59]. 

Findings suggested that 24-h PWV was associated with CSVD progression, measured by the development of new LI and cerebral microbleeds in patients with recent small subcortical infarcts, but this association was attenuated by age [32]. Additionally, changes in 24-h PWV were linked to increased blood-brain barrier (BBB) permeability in recent small subcortical infarcts, suggesting a role for arterial stiffness in vascular integrity loss [60].

Recent evidence highlights the association between arterial stiffness and subcortical infarcts with cognitive function. One article reported an association between PWV and LI in a memory clinic cohort [61]. Similarly, another article linked higher baPWV to worse cognitive function in patients with acute lacunar infarctions, establishing that elevated baPWV correlated with poorer performance in attention, abstraction, verbal memory, and orientation [62]. Interestingly, it was found that higher baPWV was significantly associated with increased risk of incident dementia in the presence of LI or WMH, but baPWV was associated with cognitive decline and dementia independent of CSVD severity [63]. On the contrary, a different study reported no association between baPWV and Mini-Mental State Examination (MMSE) scores in patients with LI [64].

While some studies supported the relationship between PWV and LI, others reported non-significant findings. One study concluded that elevated baPWV was not associated with LI, and another article found no significant relationship between cfPWV and LI [27,30].

Perivascular spaces

Several studies have explored the association between arterial stiffness, measured through PWV, and the severity of PVS, particularly in the basal ganglia (BG) and centrum semiovale (CS). Global cerebral PWV (gcPWV), measured via 4D flow MRI, was found to be associated with white matter PVS and WMH [65]. These findings also extend to stroke populations, with higher baPWV correlating with greater enlarged PVS burden (EPVS) in both BG and CS regions among patients with acute ischemic stroke [66].

In a subgroup analysis, the association of baPWV with PVS in white matter was stronger among those younger than 55 years of age. In contrast, the association with brain atrophy was more prominent among those aged 55 years and older [30]. Multiple studies have reported a consistent association between elevated cfPWV or baPWV and increased overall CSVD burden and/or basal ganglia enlarged perivascular space (BG-EPVS) severity, even among those with poorly controlled blood pressure but without a history of stroke or dementia, and individuals free of cardiovascular disease or stroke [59,67]. Similarly, another study reported that baPWV was significantly associated with the severity of BG-EPVS but not with centrum semiovale enlarged perivascular space (CS-EPVS) in patients without dementia [68]. Collectively, these findings support the hypothesis that increased arterial stiffness is associated with PVS burden, particularly in the basal ganglia, potentially through increased cerebrovascular pulsatility or endothelial dysfunction.

Cerebral microbleeds

Numerous studies have examined the link between arterial stiffness and CMB, with evidence indicating that increased PWV is associated with the presence of CMB, independent of vascular disease or prior stroke history. Increased baPWV was associated with a higher burden of CMB in cardiovascular and stroke-free individuals, as well as in patients with coronary artery disease but no prior stroke history [66,69].

More nuanced findings have emerged when considering the location of CMB. In patients with acute ischemic stroke, increased baPWV was associated with deep CMB but not with strictly lobar types. [70]. Similarly, increased cfPWV was linked to deep and mixed-location CMB in a community-dwelling cohort, with stronger associations observed among men and individuals carrying at least one APOE ε4 allele [58,67].

However, other studies have failed to demonstrate a significant relationship. No association between cfPWV and CMB was observed in cognitively healthy adults or in older adults more broadly [44,47]. Likewise, PWV was not associated with CMBs in a memory clinic cohort [61]. These mixed findings highlight the complexity of this relationship and suggest that demographic variables, comorbidities, and the specific PWV measurement techniques employed may influence observed associations.

Stroke

The studies suggest that different stroke subtypes may have distinct vascular characteristics. Patients with lacunar strokes were found to have a higher aortic PWV and central systolic blood pressure than those with other stroke subtypes [71]. Similarly, it was observed that PWV was significantly higher in patients with SVD-related stroke compared to other stroke subtypes [72]. Conversely, one study distinguished lacunar stroke from cortical stroke by identifying differences in tricuspid regurgitation velocity, blood pressure, and history of smoking, but found that PWV was similar between the two groups, suggesting different underlying mechanisms for these stroke subtypes [73].

Several studies demonstrated a significant relationship between higher baPWV and adverse stroke outcomes. One article found that the highest tertile of baPWV had nearly double the risk of poor functional outcomes, with baPWV serving as an independent prognostic value for predicting long-term functional outcomes in patients with acute cerebral infarction, regardless of stroke subtype [74]. Similarly, two articles reported significantly higher baPWV in ischemic stroke patients compared to controls [55,75]. A higher carotid-cerebral PWV (ccPWV) was also observed to be associated with severe initial stroke severity, particularly in small vessel occlusion (SVO) strokes [76]. Moreover, individuals with white coat hypertension (WCH) exhibited a higher cfPWV and higher prevalence of lacunar stroke independent of ambulatory blood pressure, suggesting PWV may play a mediating role between WCH and CSVD [77]. With regard to stroke recurrence, it was reported that higher baPWV values in patients who developed acute lacunar infarction were associated with a twofold increased risk of recurrent ischemic stroke independent of patient age, sex, and blood pressure [37].

As a convenience, all of the studies included in this review are summarized in Table 1, categorized based on study aim, sample size, design, primary findings, and CSVD markers measured. 

**Table 1:** Studies included in the review ABI: ankle-brachial index, BBB: blood-brain barrier, BP: blood pressure, baPWV: brachial-ankle pulse wave velocity, CAC: coronary artery calcification, CADASIL: cerebral autosomal dominant arteriopathy with subcortical infarcts and leukoencephalopathy, CAVI: cardio-ankle vascular index, ccPWV: carotid-cerebral pulse wave velocity, cfPWV: carotid-femoral pulse wave velocity, CSVD: cerebral small vessel disease, CMB: cerebral microbleeds, FA: fractional anisotropy, GLI: giant lacunar infarction, gcPWV: global cerebral pulse wave velocity, ICL: intra-cranial lesions, LI: lacunar infarct, MVD: microvascular dysfunction, PVS: perivascular spaces, PWV: pulse wave velocity, SVD: small vessel disease, TIA: transient ischemic attack, WMH: white matter hyperintensities.

Reference	Country	Aim of the study	Sample size	Study design	Study findings	CSVD markers
Rensma et al. (2020) [10]	Netherlands	To investigate the association between arterial stiffness, measured by cfPWV and carotid distensibility, and cognitive performance and to examine whether this association is mediated by MVD.	2,544	Cross-sectional	Higher cfPWV was associated with lower cognitive performance, partly mediated by MVD, explaining 16.2% of the effect. Carotid stiffness showed no significant association with cognitive function.	WMH, LI, CMB
Liu et al. (2020) [22]	China	To investigate whether baPWV is associated with the total CSVD score and each of its components in a healthy, asymptomatic population, and to establish baPWV cutoff values for detecting CSVD.	684	Cross-sectional	Higher baPWV correlated with increased total CSVD score and each individual marker. Optimal baPWV cutoff values were identified as 13.12 m/s for CSVD presence and 15.63 m/s for severe CSVD.	LI, WMH, CMB, PVS
Del Brutto et al. (2018) [27]	Ecuador	To assess the relationship between aortic PWV and the total CSVD score in a rural Amerindian population, as well as to evaluate individual CSVD markers such as WMH, CMB, LI, and PVS.	303	Cross-sectional	Higher aortic PWV was significantly associated with a higher total CSVD score. Individual CSVD markers, except for LI, were significantly associated with higher aortic PWV. The findings suggest a strong link between arterial stiffness and overall burden of CSVD in older adults.	LI, WMH, CMB, PVS
Zhang et al. (2020) [28]	China	To investigate the impact of aortic and carotid stiffness on carotid flow pulsatility and its association with ischemic stroke subtypes, particularly lacunar stroke.	904	Cross-sectional	Higher baPWV was significantly associated with increased odds of each CSVD marker (LI, WMH, CMB, PVS) and higher total CSVD burden. Carotid plaque presence was also associated with greater WMH volume and PVS.	LI, WMH, CMB, PVS
Kim et al. (2016) [29]	South Korea	To investigate the association between baPWV and both acute and chronic CSVD in patients with ischemic stroke or TIA.	1,282	Cross-sectional	A one standard deviation increase in baPWV was significantly associated with chronic LI, WMH, deep CMB, and acute lacunar infarctions. Higher baPWV was associated with a combined CSVD score, indicating a link between arterial stiffness and the overall burden of cerebral CSVD.	LI, WMH, CMB
Zhai et al. (2018) [30]	China	To investigate the association between arterial stiffness, measured by baPWV, and various markers of CSVD, including LI, WMH, CMB, PVS, and brain atrophy.	953	Cross-sectional	Higher baPWV was associated with severe PVS in white matter, larger WMH volume, lower brain parenchymal fraction (indicating brain atrophy), and marginally with strictly lobar CMBs but not with LI. The association of baPWV with WMH volume was consistent across age groups, while the association with brain atrophy was more significant in those aged 55 and older.	LI, WMH, CMB, PVS
Chang et al. (2023) [31]	Taiwan	To investigate whether the ABI and baPWV can reflect the severity of SVD and large artery atherosclerosis in stroke patients.	820	Cross-sectional	ABI was inversely correlated with extracranial and intracranial vessel stenosis and independently predicted moderate to severe vessel stenosis. baPWV had positive correlations with stenosis but did not independently predict severity after adjusting for confounding factors. Neither ABI nor baPWV was independently associated with SVD severity.	LI, CMB, PVS, WMH
Mena et al. (2023) [32]	Spain	To assess the association between arterial stiffness (24-h PWV) and the progression of CSVD, focusing on the development of new LI and CMB in patients with a recent small subcortical infarct.	92	Cross-sectional	Higher 24-h PWV was significantly associated with the development of new LI and CMB. The strongest predictors for new lesions were the baseline CSVD score and age. However, PWV remained an independent predictor when included in models without age.	LI, CMB, WMH, PVS
Miyagi et al. (2023) [33]	Japan	To examine the association between arterial stiffness (measured by baPWV) and CSVD markers in stroke-free individuals, considering the effects of BP status.	1,894	Cross-sectional	Higher baPWV was significantly associated with a higher prevalence of CSVD, independent of BP status. The association was observed in both hypertensive and normotensive individuals, suggesting that arterial stiffness is a critical factor for CSVD beyond BP control.	WMH, CMB, PVS, LI
Amier et al. (2021) [34]	Netherlands	To investigate the association of hypertensive exposure markers (aortic pulse wave velocity, left ventricular mass index, and mass-to-volume ratio) with CSVD and cognitive impairment using MRI.	559	Cross-sectional	Higher aortic PWV, left ventricular mass index, and left ventricle mass-to-volume ratio were associated with CSVD markers and cognitive impairment, particularly among those with cardiovascular disease. CSVD mediated the relationship between hypertensive exposure and cognitive impairment, suggesting CSVD as a pathway linking hypertension with cognitive decline.	WMH, LI, CMB, PVS
Inkeri et al. (2021) [35]	Finland	To investigate the relationship between arterial structural and functional changes, specifically carotid intima-media thickness and arterial stiffness, and CSVD in neurologically asymptomatic individuals with type 1 diabetes.	186	Cross-sectional	Higher carotid intima-media thickness was independently associated with the presence of CMB in individuals with type 1 diabetes. Arterial stiffness and carotid intima-media thickness were elevated in individuals with WMH but were not independently associated with CSVD markers after adjusting for cardiovascular risk factors.	CMB, WMH
Del Brutto et al. (2022) [36]	Ecuador	To assess the impact of aortic PWV on the progression of WMH in a cohort of older adults of Amerindian ancestry.	260	Cross-sectional	Higher PWV was significantly associated with WMH progression. The incidence rate ratio for WMH progression was 2.06 for the second tertile and 2.75 for the third tertile of PWV compared to the first tertile. The association persisted after adjusting for age, sex, and other cardiovascular risk factors, suggesting a link between arterial stiffness and CSVD.	WMH
Saji et al. (2017) [37]	Japan	To determine whether PWV predicts future ischemic stroke in patients who developed acute LI attributed to CSVD.	156	Retrospective Study	High baPWV values were associated with a twofold increased risk of recurrent ischemic stroke. Higher baPWV was significantly associated with the presence of silent LI and WMH, indicating a link between arterial stiffness and cerebral small vessel disease.	LI, WMH
Elyas et al. (2021) [38]	United Kingdom	To identify potential modifiable vascular targets for the treatment of CSVD by assessing the relationships between systemic vascular characteristics (macrovascular and microvascular function) and WMH.	112	Cross-sectional	CfPWV and carotid intima-media thickness were positively associated with WMH volume, suggesting arterial stiffness and atherosclerosis contribute to CSVD. Microvascular reactivity, urinary albumin excretion, and adjusted cerebral resistance index were also associated with WMH volume, independent of age and sex, highlighting therapeutic targets.	WMH
Moreton et al. (2021)[39]	United Kingdom	To investigate the relationship between vascular measures, including cfPWV, and the progression of subcortical hyperintensities in CADASIL patients over a two-year period.	22	Cohort	Baseline cfPWV predicted the progression of subcortical hyperintensity volume at follow-up, indicating that arterial stiffness contributes to disease progression in CADASIL. There was a significant increase in the number of LI and CMB over the study period, along with a decline in cerebral blood flow.	LI, CMB, WMH
Pase et al. (2016) [40]	United States	To examine the association between aortic stiffness, measured by cfPWV, cognitive performance, and markers of subclinical brain injury in young to middle-aged adults.	3,207	Cross-sectional	Higher cfPWV was associated with poorer processing speed, larger lateral ventricular volumes, and a higher burden of WMH. Associations varied by age; cfPWV was associated with ventricular volume in younger adults (30-45 years) and with WMH and cognition in middle-aged adults (45-65 years).	WMH, PVS
Armstrong et al. (2024) [41]	USA	To determine the association of structural versus load-dependent large artery stiffness mechanisms with cerebrovascular damage measured as white matter lesion volume and cortical atrophy in cognitively normal middle-aged and older adults.	128	Cross-sectional	Greater structural aortic stiffness was linked to higher total and periventricular white matter lesion volume and lower cortical thickness, while load-dependent stiffness showed no significant association with cerebrovascular damage.	WMH, PVS
Zhai et al. (2020) [42]	China	To investigate the associations of carotid atherosclerosis, dilation, and stiffness with imaging markers of CSVD (LI, WMH, CMB, PVS) and their spatial distribution in a community-based sample.	1,051	Cross-sectional	Carotid dilation was significantly associated with lacunes, larger WMH volume, and dilated PVS, independent of carotid intima-media thickness and plaque presence. CfPWV was not significantly associated with CSVD markers after adjusting for confounders.	LI, WMH, CMB, PVS
Haidegger et al. (2023) [43]	Austria	Investigate the association between PWV and its circadian changes on brain morphology and cognitive function in community-dwelling elderly individuals.	84	Cross-sectional	PWV was significantly related to reduced total brain volume, independent of blood pressure. Night-time PWV was associated with global brain atrophy, while no association was found between PWV and cognitive impairment or small vessel disease markers.	WMH
Gustavsson et al. (2015) [44]	Sweden	Investigate if arterial stiffness influences the presence of CMB, WMH, and cognitive function in a population of cognitively healthy elderly individuals.	208	Cross-sectional	CMB were present in 12% of participants and WMH in 31%. No association was found between CMB and arterial stiffness. However, arterial stiffness was positively associated with WMH, though this association diminished after adjustments for cardiovascular risk factors. WMH showed a weak negative association with cognitive performance in one test of attention.	WMH, CMB
Liu et al. (2020) [45]	United States and China	To investigate the associations between arterial stiffness (measured by cfPWV) and cerebrovascular health, including small vessel disease (measured by WMH), intracranial atherosclerosis, and cerebral blood flow in older hypertensive males.	200	Cross-sectional	Higher cfPWV was significantly associated with lower cerebral blood flow, increased risk of intracranial stenotic plaques, and greater WMH volume. Endothelial dysfunction was independently associated with reduced cerebral blood flow but not with WMH or intracranial plaques. These findings suggest that both arterial stiffness and endothelial dysfunction contribute to impaired cerebrovascular health in hypertensive patients.	WMH
Azahar et al. (2023) [46]	Japan	To investigate the independent associations of arterial stiffness (measured by baPWV) and atherosclerotic burden (measured by CAC) with brain structural changes in a Japanese population.	686	Cross-sectional	Higher baPWV was associated with lower Alzheimer’s disease signature volume, suggesting a link between arterial stiffness and brain atrophy. Higher CAC scores were independently associated with increased WMH, indicating a relationship between atherosclerotic burden and brain vascular damage.	WMH
Palta et al. (2019) [47]	United States	To examine the association between central arterial stiffness, measured by cfPWV, and brain structural changes and cognitive performance among older adults.	3,703	Cross-sectional	Higher cfPWV was associated with increased WMH volume, reduced total brain and Alzheimer’s disease signature region volumes, and poorer performance in executive function and general cognition. Higher central pulse pressure also correlated with cognitive decline, specifically in language and executive function domains.	WMH, LI, CMB
Bown et al. (2021) [48]	United States	The study aimed to determine if baseline aortic stiffness, measured by aortic PWV, is related to longitudinal changes in cerebral gray matter and white matter in older adults.	278	Cross-sectional	Higher baseline aortic PWV was associated with greater decreases in gray matter volume in the hippocampus and occipital lobe and greater increases in WMH volume in the temporal lobe over time. The associations were stronger in participants without the APOE ε4 allele.	WMH
van Sloten et al. (2016) [49]	Iceland, The Netherlands, and the United States	To examine the association between arterial stiffness (measured by cfPWV) and depressive symptoms, and whether CSVD mediates this relationship in older adults.	2058	Cross-sectional	Higher cfPWV was associated with more depressive symptoms. The relationship was partly mediated by WMH volume and subcortical infarcts, suggesting that CSVD contributes to the link between arterial stiffness and depression. Other CSVD markers (CMB and PVS) did not mediate the association between cfPWV and depressive symptoms.	LI, WMH, CMB, PVS
Funck et al. (2021) [50]	Germany	To investigate the association between arterial stiffness, measured by cfPWV, and brain structural changes, specifically WMH, in middle-aged adults with type 2 diabetes over a five-year period.	150	Cohort	Higher cfPWV at baseline was significantly associated with increased WMH volume at follow-up. Participants with type 2 diabetes exhibited a greater increase in WMH volume compared to controls, suggesting that arterial stiffness may contribute to microvascular brain damage in diabetes. A significant association was found between increased cfPWV and reduced performance in executive function tests over the five-year follow-up period.	WMH
Wei et al. (2020) [51]	United States	To examine the association between aortic stiffness (measured by cfPWV) and white matter microstructural integrity using diffusion tensor imaging in older adults.	1,484	Cross-sectional	Higher cfPWV was associated with lower FA and higher mean diffusivity across the brain, indicating reduced white matter microstructural integrity. The associations were stronger in participants with higher WMH volumes, suggesting a link between increased arterial stiffness and greater microvascular brain damage. cfPWV remained a significant predictor of lower white matter integrity even after adjusting for WMH volume and other covariates.	WMH, FA
Cooper et al. (2022) [52]	United States	To investigate the relationship between postural changes in blood pressure and aortic stiffness (cfPWV) with hypertension-mediated organ damage in middle-aged adults.	3,495	Cross-sectional	Higher cfPWV was associated with increased white matter free water content, indicating subclinical cerebral injury. Significant sex-specific differences were observed in the association between cfPWV and kidney damage (urinary albumin-creatinine ratio), with stronger associations in men.	LI, WMH
Suzuki et al. (2021) [53]	United States	To investigate the association between CAC assessed years earlier and subtle white matter injury in the brain, using diffusion tensor imaging measures in asymptomatic middle-aged men.	1,052	Observational	Higher CAC scores were associated with lower FA (indicating white matter injury) in men but not in women, suggesting CAC as a potential predictor for subtle brain changes in men.	FA
Meyer et al. (2020) [54]	United States	To investigate the association between central arterial stiffness, measured by cfPWV, and retinal vessel calibers, specifically the central retinal arteriolar equivalent and central retinal venular equivalent, as a proxy for cerebral microvascular health.	1,706	Cross-sectional	Higher cfPWV was associated with wider central retinal venular equivalent, particularly in individuals without hypertension. No significant association was found between cfPWV and central retinal arteriolar equivalent narrowing. The association between cfPWV and central retinal venular equivalent widening was stronger in individuals with type 2 diabetes.	Retinal Vessel Caliber
Lee et al. (2016) [55]	South Korea	To investigate the relationship between ABI, baPWV, and markers of cerebrovascular disease in ischemic stroke patients, aiming to differentiate between large artery disease and SVD.	121	Retrospective	baPWV was significantly higher in the stroke group vs. controls. Higher baPWV was associated with small vessel disease, including lacunar infarctions and leukoaraiosis, with a significant correlation. The study concluded that baPWV is a reliable marker for detecting small vessel disease, while ABI is more indicative of large artery disease.	LI
Sato et al. (2020) [56]	Japan	To identify and compare risk factors for infarct expansion in patients with LI and GLI, exploring different pathophysiological mechanisms for the two subtypes.	154	Retrospective	Higher CAVI and uric acid levels were significantly associated with infarct expansion in LI patients. Higher low-density lipoprotein cholesterol and lower body mass index were associated with infarct expansion in GLI patients, highlighting distinct mechanisms for infarct expansion between LI and GLI.	LI
Shan et al. (2016) [57]	China	To examine the relationship between aortic compliance (measured by aortic arch PWV) and brachial artery endothelial function (measured by flow-mediated dilation) with CSVD in patients with type 2 diabetes.	62	Cross-sectional	Higher aortic arch PWV was significantly associated with lacunar brain infarcts. Flow-mediated dilation was significantly associated with periventricular WMH, but not with deep WMH or LI.	LI, WMH
Tabata et al. (2017) [58]	Japan	To investigate the association between baPWV and asymptomatic ICL in coronary artery disease patients without a history of stroke.	149	Cross-sectional	Higher baPWV was significantly associated with the presence of ICL, with baPWV acting as an independent predictor. Patients with ICL had worse cardiovascular outcomes, with a higher incidence of adverse events over the follow-up period (mean: 707 days).	LI, CMB
Riba-Llena et al. (2018) [59]	Spain	To assess whether arterial stiffness, measured by cfPWV, is associated with the load of CSVD and its individual markers (LI, WMH, CMB, PVS).	782	Cross-sectional	Higher cfPWV was associated with greater CSVD load, particularly with the presence of LI and basal ganglia enlarged PVS. Associations were stronger in participants with poorly controlled blood pressure.	LI, WMH, CMB, PVS
Romo et al. (2024) [60]	Spain	To examine the relationship between changes in PWV and BBB permeability in patients with a recent small subcortical infarct, assessing whether increased arterial stiffness is linked to BBB disruption.	29	Cross-sectional	Increased PWV over time was significantly correlated with higher BBB permeability, suggesting arterial stiffness may contribute to BBB disruption in patients with CSVD. This persisted after adjusting for confounding factors, indicating PWV changes may play a more critical role than blood pressure alone.	LI
Robert et al. (2022) [61]	Singapore	To examine the association of carotid artery stiffness with markers of CSVD, cognitive impairment, and dementia subtypes in a memory clinic cohort.	272	Cross-sectional	Carotid artery stiffness measures were significantly associated with increased WMH and LI. Higher carotid stiffness was linked to vascular dementia but not Alzheimer’s disease. Cognitive impairments were partially mediated by the presence of CSVD markers.	LI, WMH, CMB
Huang et al. (2020) [62]	China	To explore the association between ABI, baPWV, and mild cognitive impairment in patients with acute lacunar infarction.	103	Cohort	Higher baPWV and lower ABI were correlated with worse performance in the attention, abstraction, delayed verbal memory, and orientation tasks, but not in naming or ability to language task.	LI
Yamagishi et al. (2025) [63]	Japan	To investigate the predictive value of baPWV for dementia and cognitive decline in patients with CSVD.	478	Cross-sectional	Higher baPWV was significantly associated with increased risk of incident dementia after adjusting for confounding factors. Higher baPWV was also associated with greater cognitive decline over a three-year period.	LI, WMH
Nakamori et al. (2022) [64]	Japan	To investigate the association between ABI, baPWV, and cognitive decline in patients with lacunar infarction.	176	Cross-sectional	Lower ABI was significantly associated with lower Mini-Mental State Examination scores, indicating cognitive decline. baPWV was not independently associated with cognitive decline after adjusting for confounders. The findings suggest that peripheral arterial disease (low ABI) may be a stronger indicator of cognitive impairment compared to arterial stiffness (baPWV) in lacunar infarction patients.	LI
Björnfot et al. (2024) [65]	Sweden	To examine the relationship between cerebral arterial stiffness, measured by gcPWV, and markers of CSVD.	190	Cross-sectional	Higher gcPWV was significantly associated with greater volumes of WMH and PVS in white matter, suggesting that increased cerebral arterial stiffness is linked to CSVD. These associations remained significant after adjusting for potential confounders, indicating that gcPWV might be an early marker of CSVD.	LI, WMH, PVS
Bae et al. (2021) [66]	South Korea	To investigate the relationship between arterial stiffness, measured using baPWV, and the presence of cerebral small vessel disease markers, including PVS and CMB, in acute ischemic stroke patients.	854	Cross-sectional	Higher baPWV was associated with increased severity of PVS at the basal ganglia and centrum semiovale levels. Deep CMB were also significantly related to higher baPWV, while strictly lobar CMB were not. Results suggest elevated baPWV associated with hypertensive arteriopathy affecting cerebral small vessels.	PVS, CMB
Kinjo et al. (2016) [67]	Japan	This study aimed to investigate the relationship between ABI, baPWV, and CMB.	990	Cross-sectional	Higher ABI and baPWV were independently associated with the presence of CMB. The combination of high ABI and high baPWV was strongly associated with CMB, suggesting a link between increased arterial stiffness and microvascular brain damage.	CMB, WMH
Kinjo et al. (2024) [68]	Japan	To investigate the relationship between baPWV and the severity of enlarged PVS in participants without dementia.	74	Cross-sectional	High baPWV was independently associated with increased severity of basal ganglia enlarged PVS, but not with centrum semiovale enlarged PVS. Older age was associated with both basal ganglia enlarged PVS and centrum semiovale enlarged PVS.	PVS
Song et al. (2014) [69]	South Korea	To investigate the relationship between arterial stiffness, measured by baPWV, and the presence and location of CMB in patients with non-cardioembolic acute ischemic stroke.	1,137	Cross-sectional	Higher baPWV was independently associated with deep or infratentorial CMB but not with strictly lobar CMB, suggesting a link between arterial stiffness and CMB in deep brain regions.	CMB
Romero et al. (2021) [70]	United States	To investigate the association between aortic stiffness, measured by cfPWV, and the prevalence of CMB in a community-dwelling cohort from the Framingham Heart Study.	3,798	Cross-sectional	Higher cfPWV was associated with increased odds of deep and mixed-location CMB, with associations more pronounced in men and in participants with at least one APOE ε4 allele. Associations were modest after adjusting for vascular risk factors and treatments.	CMB
Wohlfahrt et al. (2014) [71]	Czech Republic	To investigate the impact of aortic and carotid stiffness on carotid flow pulsatility and its association with ischemic stroke subtypes, particularly lacunar stroke.	184	Cross-sectional	Patients with lacunar stroke had higher aortic PWV and central systolic blood pressure than those with other stroke subtypes. Aortic stiffness was a significant determinant of resistive index, indicating that aortic stiffening may contribute to lacunar stroke pathogenesis by increasing pressure transmission to cerebral arterioles.	LI
Webb et al. (2023) [72]	United Kingdom	To examine differences in intermediate cardiovascular phenotypes, such as arterial stiffness (PWV), cerebral arterial pulsatility, and beat-to-beat blood pressure variability, across different ischemic stroke etiologies, including large artery stroke, cardioembolic, SVD, and undetermined causes.	909	Cross-sectional	PWV and cerebral pulsatility index were significantly higher in patients with SVD compared to other stroke subtypes, suggesting a link between arterial stiffness and SVD. The relationship between age and arterial stiffness was stronger in patients with SVD, indicating accelerated vascular aging in this group.	LI
Muscari et al. (2016) [73]	Italy	To identify risk factors for lacunar strokes with visible lesions on CT scan, focusing on clinical characteristics, echocardiographic parameters, and cfPWV as a marker of large-artery stiffness.	106	Cross-sectional	Lacunar stroke patients had lower tricuspid regurgitation velocity, higher systolic blood pressure, a higher prevalence of ever-smokers, and a lower prevalence of atrial fibrillation compared to cortical stroke patients. CfPWV and left ventricular mass index were similar between the two groups, suggesting different underlying mechanisms for lacunar and cortical strokes.	LI
Kim et al. (2014) [74]	South Korea	To determine if baPWV has prognostic value for predicting functional outcomes in acute cerebral infarction and if this prognostic value differs by stroke subtype.	1,091	Retrospective	Higher baPWV was significantly associated with poor functional outcomes, with patients in the highest tertile of baPWV facing almost double the risk of poor outcomes. BaPWV showed independent prognostic value for outcomes across stroke subtypes.	LI
Saji et al. (2015) [75]	Japan	To compare the utility of three arteriosclerotic indicators, ABI, baPWV, and CAVI, in assessing arterial stiffness and their relationship to acute ischemic stroke subtypes, including large artery atherosclerosis and small artery disease.	842	Cross-sectional	baPWV and CAVI were significantly higher in patients with ischemic stroke subtypes compared to controls, indicating increased arterial stiffness. The cutoff value for baPWV for detecting acute ischemic stroke was 18.3 m/s.	LI, WMH
Fu et al. (2019) [76]	China	To assess the association between cerebral arterial stiffness (measured by ccPWV) and the initial severity of acute ischemic stroke.	842	Cross-sectional	Higher ccPWV was significantly associated with severe initial stroke severity, with an optimal cutoff value of 6.87 m/s. ccPWV was more strongly correlated with the severity of small-vessel occlusion compared to other stroke subtypes, indicating a link between cerebral arterial stiffness and microvascular damage. The odds of severe initial stroke severity were significantly higher for patients with ccPWV > 6.87 m/s.	LI
Saunders et al. (2022) [77]	United Kingdom	To investigate the association between white-coat hypertension/effect, arterial stiffness, and the prevalence of lacunar stroke in patients with a recent diagnosis of TIA or lacunar stroke.	842	Cross-sectional	Patients with white-coat hypertension/effect had significantly higher PWV compared to those with target BP. White-coat hypertension/effect was independently associated with a higher prevalence of lacunar stroke, with an odds ratio of 9.6.	LI

Discussion

This scoping review aimed to determine whether there was an association between CSVD and PWV. Our search revealed a general pattern of association between several CSVD markers and PWV, with some articles reporting no association due to potential confounders, specific markers, or the use of particular PWV measurements. 

PWV-CSVD General Association Findings 

Most papers have reported an association between PWV and total CSVD burden and individual CSVD markers [33,59,77]. Higher baPWV was associated with a significantly higher prevalence of CSVD, independent of blood pressure (BP) status [33,77]. This suggests that arterial stiffness may be a stronger determinant of CSVD risk than BP alone [33,77]. This may be because BP is a dynamic measure that can fluctuate throughout the day due to stress, medication, or environmental factors. At the same time, PWV reflects long-term cumulative damage to the vascular system and directly reflects the arterial wall's structural rigidity and mechanical properties [78]. Given the high cost of imaging screening for CSVD, PWV may serve as a practical screening tool to identify individuals at risk, facilitating earlier intervention. Future research should investigate the predictive value of PWV for tracking CSVD severity and informing vascular risk management strategies.

PWV-CSVD and Cardiovascular Comorbidities 

A key theme across studies is the presence of arterial stiffness in populations with pre-existing cardiovascular comorbidities. Elevated aortic PWV and baPWV were associated with lacunar infarcts in individuals with T2DM and CAD, respectively [57,58]. Moreover, in patients with T2DM, the increase in WMH volume was greater compared to those without T2DM [50]. These findings suggest that cardiovascular comorbidities may contribute to the development and progression of CSVD through their impact on arterial stiffness. Conditions such as T2DM and CAD promote endothelial dysfunction, inflammation, and impaired vascular elasticity, all of which can accelerate arterial stiffening and disrupt cerebral microvascular integrity [79]. These findings highlight the possibility that arterial stiffness serves as a link between cardiovascular disease and cerebrovascular damage, reinforcing the need for targeted management of hypertension, diabetes, and CAD to mitigate CSVD progression.

PWV-CSVD and Stroke Risk

Our studies found that PWV may also shape recovery and long-term functional outcomes. In one study, it was reported that patients in the highest tertile baPWV had nearly twice the risk of poor functional outcomes, with baPWV serving as an independent prognostic marker for predicting long-term functional outcomes in acute cerebral infarction, regardless of stroke subtype [74]. Given that CSVD is a major contributor to cerebral infarcts and stroke risk, these findings suggest that arterial stiffness may exacerbate cerebrovascular damage, aggravating the effects of CSVD and therefore impairing the brain’s ability to recover following a stroke. Two studies reported higher baPWV in ischemic stroke patients compared to controls, further supporting the role of arterial stiffness as a marker of cerebrovascular dysfunction [55,75]. Additionally, elevated ccPWV was associated with more severe initial stroke presentations, particularly in SVO strokes [76]. This reinforces the notion that arterial stiffness may directly contribute to microvascular damage, worsening stroke severity and increasing the risk of long-term functional impairment. 

PWV not only influences the likelihood of an initial stroke, but also appears to perpetuate a cycle of recurrent ischemic events and CSVD advancement. Higher baPWV values were associated with a twofold increased risk of recurrent ischemic stroke in lacunar infarct patients [37]. This highlights arterial stiffness as a key determinant of stroke prognosis in patients with CSVD. The interplay between arterial stiffness and stroke recurrence suggests a cycle in which increased vascular rigidity may contribute to cerebral perfusion deficits and small vessel damage, which in turn predisposes individuals to recurrent strokes, CSVD progression, and worsening cerebrovascular outcomes. Understanding this cycle may offer new opportunities for intervention, targeting PWV as a modifiable risk factor in CSVD management. 

PWV-CSVD in Cognitive Impairment and Depression

Several studies have investigated the relationship between PWV and cognitive impairment or depression in the context of CSVD. Elevated cfPWV has been associated with greater depressive symptoms, partially mediated by WMH volume and subcortical infarcts, though not by CMB or visible PVS [47,48]. Higher cfPWV and aortic PWV have also been linked to structural brain damage and progressive hippocampal atrophy, respectively, both of which contribute to cognitive decline [40]. Additionally, increased cfPWV has been correlated with poorer executive function in midlife adults, with WMH volume again serving as a partial mediator [63]. These findings suggest that ischemic CSVD markers, particularly WMH and subcortical infarcts, may play a more prominent role in depression and cognitive impairment than hemorrhagic lesions such as CMBs.

However, the mediating role of CSVD in these associations requires nuanced interpretation. Notably, higher baPWV has been shown to predict increased dementia incidence and accelerated cognitive decline even after adjusting for baseline CSVD burden [80]. This supports the growing recognition that arterial stiffness may directly contribute to neurodegeneration through mechanisms such as amyloid accumulation, chronic cerebral hypoperfusion, and BBB disruption, as demonstrated in a longitudinal study adjusting for CSVD markers [40].

The stage of vascular pathology may also influence these associations. For instance, in a younger cohort, no significant relationship was found between cfPWV and depression or memory performance, and WMH volume did not serve as a mediator [81]. These results likely reflect earlier disease stages, where vascular effects may be limited to executive dysfunction via frontal-subcortical pathway disruption, while memory deficits and depressive symptoms tend to emerge later alongside neurodegenerative changes or more advanced vascular injury affecting deeper brain regions such as the basal ganglia [81].

PWV-CSVD Confounding Factors

Some studies noted no association between CSVD and PWV after adjusting for age and arterial risk factors such as hypertension and weight [31,42-44]. As we age, our elastin fibers in the arterial wall decay, losing their function and shifting load bearing to the stiffer collagen fibrils, directly causing increased arterial stiffness [82]. Over time, this exposes the brain microvasculature to abnormal blood flow and contributes to the severity and development of CSVD [20]. As such, a predominantly older population can make the PWV-CSVD association appear more strongly than it is, and a younger patient population could reduce the frequency of small vessel disease. Furthermore, prior research has established that vascular risk factors such as blood pressure and obesity can increase arterial stiffening [83,84] and that lifestyle interventions and antihypertensive medications can decrease PWV [83,85,86]. The studies also acknowledged that a larger study population with a diverse age group and vascular characteristics could better address this limited generalizability. 

One study found baPWV was more strongly associated with PVS in white matter in younger adults (<55 years) than older (>55 years) [30]. Earlier research has shown that PVS is one of the earliest signs of CSVD [84], and the stronger PWV-PVS association in younger adults aligns with findings from other studies [87]. Consequently, assessing baPWV in younger individuals with cardiovascular risk factors may provide a valuable opportunity for early detection of CSVD-related changes. Identifying these patients earlier could enable timely interventions such as lifestyle modifications, exercise, and antihypertensive treatments to slow or prevent CSVD progression. Some studies found that the association between WMH and baPWV stayed consistent across age groups [30,40,41], suggesting that baPWV could also assess long-term CSVD risk in both younger and older adults.

Differences in PWV measurement outcomes can be attributed to variations in the patient population and underlying conditions. In individuals with T1DM, cfPWV showed a stronger association with CSVD markers such as WMH and CMB, while baPWV did not demonstrate a significant correlation [35]. Similarly, in acute ischemic stroke patients, no association was found between baPWV and CSVD severity [31]. One article postulates that baPWV may not be a good indicator for predicting vessels in patients with a higher baPWV baseline, which would help explain the lack of association in AIS or T1D patients [31]. 

Regional and Structural Considerations in PWV-CSVD Association 

Structural and regional differences can account for changes in PWV-CSVD association. Early indicators of deterioration in white matter microstructure include fractional anisotropy, mean diffusivity, and free water content. Two studies have shown that higher cfPWV was significantly associated with lower values for each of these parameters [51,53]. Increased PWV can cause endothelial dysfunction and disruption of the BBB, increasing susceptibility to ischemic injury in white matter tracts and permitting excess fluid leakage into the brain parenchyma [88]. However, in middle-aged adults, one article found no association between cfPWV and free water [52], and another article found a negative association in middle-aged men [53]. However, these differences can be attributed to the study's population differences. These findings suggest that identifying white matter structural injury indicators could provide an early window for intervention before severe neurological symptoms and structural lesions manifest.

The baPWV has been linked to the presence of BG-EPVS, but not CS-EPVS in individuals without dementia [68]. The BG is primarily supplied by small arteries originating directly from major cerebral arteries, making them more susceptible to changes in arterial stiffness, while the CS receives blood supply from larger, medullary arteries, potentially rendering the CS less sensitive to such changes [89]. Several articles found that PWV was associated with deep CMBs, but not strictly lobar CMBs [29,66,69]. Deep CMBs are typically associated with hypertensive arteriopathy, a condition linked to arterial stiffness, whereas lobar CMBs are often associated with cerebral amyloid angiopathy (CAA), a pathology primarily driven by amyloid-β deposition and less directly linked to vascular stiffness [90]. Increased cfPWV was found to be associated with deep CMBs and mixed-location CMBs, with the association being stronger in APOE4 carriers [70]. This may be because APOE ε4 carriers may exhibit increased vascular fragility or impaired cerebrovascular autoregulation, making them more susceptible to both amyloid pathology and microvascular damage from arterial stiffness [91]. These results highlight the need to explore cerebral regional vulnerabilities to arterial stiffness further. 

Strengths

This scoping review is strengthened by its broad and inclusive selection criteria, aimed at reflecting the real-world complexity of CSVD research. Studies using a wide range of imaging modalities (MRI, CT, MRA, and CTA) were included to enhance the generalizability of the findings, accounting for variability in diagnostic practices across settings and helping ensure that associations between PWV and CSVD are not dependent on a single modality’s specificity and sensitivity. Furthermore, studies were not excluded based on the type of PWV measured (e.g., baPWV, cfPWV, aortic PWV, 24-h PWV), which allows for a more comprehensive understanding of how arterial stiffness, measured across different vascular territories, relates to CSVD. Studies involving patients with comorbidities were also not excluded, providing insight into populations with conditions such as hypertension, diabetes mellitus, and cardiovascular disease, which are factors known to be linked to arterial stiffness and CSVD. By examining the heterogeneity across study methodologies and patient populations, this review offers a nuanced literature synthesis and provides meaningful insight into the multifactorial relationship between PWV and CSVD.

Limitations

Several limitations within this scoping review are to be acknowledged. Firstly, the review limited the search to only articles in English. As part of the methods, all techniques were included for measuring arterial stiffness using PWV techniques to understand its relationship with CSVD comprehensively. By definition, arterial stiffness was measured in varying vascular territories (central and/or peripheral), potentially exhibiting different sensitivities, which could affect the presence or lack of association with the factors studied. 

Furthermore, while many studies employed statistical adjustments for known confounding variables such as age, study populations, or other comorbidities, these models remain inherently limited. Relevant factors such as systemic inflammation, endothelial dysfunction, or genetic differences, among others, are often unmeasured, hard to quantify, or challenging to standardize across cohorts, thus limiting the generalizability of the findings. Additionally, the review encompassed many study types, with most studies being cross-sectional, limiting causality. These studies also had different protocols for follow-up durations or used different assessments to measure specific outcomes, further complicating interpretation. 

Studies also used different criteria to define and quantify CSVD markers, such as various size/color assessments (volume/color differences vs. visual rating score), size criteria thresholds, severity classification, detection method (MRI field strength subtypes, CT, resolution, and slice thickness), or even location sensitivity (grouping vs. stratifying). Finally, the absence of a minimum sample size requirement across studies amplifies these confounding factors' impact, affecting the robustness of the conclusions.

Future research

Although growing evidence supports the link between arterial stiffness, as measured by PWV, and CSVD, several gaps remain in the literature. Most of the studies examined are cross-sectional, limiting the ability to establish causality. Future studies should focus on longitudinal research to help determine whether elevated PWV directly contributes to CSVD progression or is simply an indicator of aging-related vascular changes. Research confirming a link between increased PWV and CSVD progression can support the use of arterial stiffness as a key strategy for early intervention of CSVD and its associated conditions, such as stroke and cognitive impairment.

Additionally, more research is needed to determine the effectiveness of early intervention strategies in slowing CSVD progression. While antihypertensive therapy, regular exercise, and dietary modifications have been proposed as potential approaches to improving vascular function, their direct influence on CSVD is not clear. Future studies should assess whether lowering PWV through these methods leads to measurable reductions in CSVD markers. Understanding the extent to which these interventions can modify disease progression will be essential for improving long-term cerebrovascular health.

Sex-specific and racial/ethnic differences in PWV-CSVD associations also need to be further explored. Men and women may exhibit different cerebrovascular responses to arterial stiffness, yet many studies conducted do not stratify findings by sex. Similarly, it is possible that racial and ethnic disparities in vascular health may influence CSVD progression. Accounting for these factors is critical for a comprehensive understanding of the relationship between arterial stiffness and CSVD and for developing targeted prevention and treatment modalities amongst diverse populations.

While the use of diverse measurement and imaging techniques across studies may enhance the generalizability of findings and contribute to a more comprehensive understanding of the relationship between PWV and CSVD, the lack of standardized assessment methods also poses a challenge in PWV-CSVD research. Differences in measurement techniques, such as cfPWV and baPWV, may yield varying results depending on the specific vascular segments assessed and the level of measurement precision. Imaging techniques also differ, with some studies using MRI and others using CT, CTA, or MRA. This makes it difficult to accurately compare results because variations in sensitivity and specificity among these imaging techniques may affect the detection of CSVD markers. Standardizing diagnostic criteria and imaging protocols will be necessary to improve the reliability and reproducibility of future research. Addressing these gaps will enhance our understanding of the relationship between arterial stiffness and CSVD and help guide clinical strategies to preserve cerebrovascular health.

## Conclusions

These findings are consistent with the potential utility of PWV for the possible earlier prediction of CSVD and possibly assessing its likely severity and progression, provided that suitable standardization of measurement methods and further longitudinal research and assessments are done. The association between PWV and cognitive decline, stroke risk, and functional recovery further underscores PWV in a potential role as a predictive marker in CSVD management. Identifying PWV as a potentially modifiable risk factor emphasizes the importance of early detection strategies, lifestyle interventions, and cardiovascular risk management to mitigate CSVD progression and its related outcomes.

## Appendices

Full search strings used in this review from each of the four databases are provided in the following.  

EMBASE

**Table 2:** Search strings used in EMBASE

Search string used in EMBASE		
#1	'basal ganglion hemorrhage'/exp OR 'brain hematoma'/exp OR 'brain hemorrhage'/exp OR 'brain infarction'/exp OR 'brain ischemia'/exp OR 'brainstem stroke'/exp OR 'cardioembolic stroke'/exp OR 'ischemic stroke'/exp OR 'lacunar stroke'/exp OR 'cerebrovascular malformation'/exp OR 'hypophysis apoplexy'/exp OR 'lipohyalinosis'/exp OR 'melas syndrome'/exp OR 'posterior reversible encephalopathy syndrome'/exp OR 'occlusive cerebrovascular disease'/exp OR 'vascular depression'/exp OR 'vertebrobasilar insufficiency'/exp	533739
#2	'brain angiopathy':ab,ti,kw OR 'brain circulation failure':ab,ti,kw OR 'brain vascular disease':ab,ti,kw OR 'brain vasculopathy':ab,ti,kw OR 'cerebral angiopathy':ab,ti,kw OR 'cerebral small vessel disease':ab,ti,kw OR 'cerebral small vessel diseases':ab,ti,kw OR 'cerebral vascular disease':ab,ti,kw OR 'cerebral vascular disorder':ab,ti,kw OR 'cerebral vascular disturbance':ab,ti,kw OR 'cerebral vascular lesion':ab,ti,kw OR 'cerebral vasculopathy':ab,ti,kw OR 'cerebro-vascular damage':ab,ti,kw OR 'cerebro-vascular disease':ab,ti,kw OR 'cerebro-vascular disorder':ab,ti,kw OR 'cerebro-vascular disturbance':ab,ti,kw OR 'cerebro-vascular lesion':ab,ti,kw OR 'cerebro-vascular pathology':ab,ti,kw OR 'cerebro-vascular syndrome':ab,ti,kw OR 'cerebro-vasculopathy':ab,ti,kw OR 'cerebroangiopathy':ab,ti,kw OR 'cerebrovascular damage':ab,ti,kw OR 'cerebrovascular disorder':ab,ti,kw OR 'cerebrovascular disorders':ab,ti,kw OR 'cerebrovascular disturbance':ab,ti,kw OR 'cerebrovascular lesion':ab,ti,kw OR 'cerebrovascular pathology':ab,ti,kw OR 'cerebrovascular syndrome':ab,ti,kw OR 'cerebrovasculopathy':ab,ti,kw OR 'cerebrovascular disease':ab,ti,kw OR 'basal ganglia cerebrovascular disease':ab,ti,kw OR 'basal ganglia haemorrhage':ab,ti,kw OR 'basal ganglia hemorrhage':ab,ti,kw OR 'basal ganglion cerebrovascular disease':ab,ti,kw OR 'basal ganglion haemorrhage':ab,ti,kw OR 'cerebrovascular disease, basal ganglion':ab,ti,kw OR 'haemorrhage, basal ganglion':ab,ti,kw OR 'hemorrhage, basal ganglion':ab,ti,kw OR 'basal ganglion hemorrhage':ab,ti,kw OR 'haemorrhage, putamen':ab,ti,kw OR 'hemorrhage, putamen':ab,ti,kw OR 'putamen haematoma':ab,ti,kw OR 'putamen haemorrhage':ab,ti,kw OR 'putamen hematoma':ab,ti,kw OR 'putamen hemorrhage':ab,ti,kw OR 'putaminal haematoma':ab,ti,kw OR 'putaminal haemorrhage':ab,ti,kw OR 'putaminal hematoma':ab,ti,kw OR 'putaminal hemorrhage':ab,ti,kw OR 'brain haematoma':ab,ti,kw OR 'cerebral haematoma':ab,ti,kw OR 'cerebral hematoma':ab,ti,kw OR 'haematoma, brain':ab,ti,kw OR 'hematoma, brain':ab,ti,kw OR 'interhemispheric haematoma':ab,ti,kw OR 'interhemispheric hematoma':ab,ti,kw OR 'intracerebral haematoma':ab,ti,kw OR 'intracerebral hematoma':ab,ti,kw OR 'intracranial haematoma':ab,ti,kw OR 'intracranial hematoma':ab,ti,kw OR 'brain hematoma':ab,ti,kw OR 'acute subdural haematoma':ab,ti,kw OR 'acute subdural hematoma':ab,ti,kw OR 'chronic subdural haematoma':ab,ti,kw OR 'chronic subdural haematomata':ab,ti,kw OR 'chronic subdural hematoma':ab,ti,kw OR 'chronic subdural hematomata':ab,ti,kw OR 'haematoma, subdural':ab,ti,kw OR 'haematoma, subdural, acute':ab,ti,kw OR 'haematoma, subdural, chronic':ab,ti,kw OR 'haematoma, subdural, intracranial':ab,ti,kw OR 'haemorrhage, subdural':ab,ti,kw OR 'haemorrhagic pachymeningitis':ab,ti,kw OR 'hematoma, subdural':ab,ti,kw OR 'hematoma, subdural, acute':ab,ti,kw OR 'hematoma, subdural, chronic':ab,ti,kw OR 'hematoma, subdural, intracranial':ab,ti,kw OR 'hemorrhage, subdural':ab,ti,kw OR 'hemorrhagic pachymeningitis':ab,ti,kw OR 'intracranial subdural haematoma':ab,ti,kw OR 'intracranial subdural haematomas':ab,ti,kw OR 'intracranial subdural haematomata':ab,ti,kw OR 'intracranial subdural hematoma':ab,ti,kw OR 'intracranial subdural hematomas':ab,ti,kw OR 'intracranial subdural hematomata':ab,ti,kw OR 'pachymeningiosis haemorrhagica interna':ab,ti,kw OR 'pachymeningitis haemorrhagica':ab,ti,kw OR 'subdural bleeding':ab,ti,kw OR 'subdural haematoma':ab,ti,kw OR 'subdural haemorrhage':ab,ti,kw OR 'subdural hemorrhage':ab,ti,kw OR 'subepidural haematoma':ab,ti,kw OR 'subepidural hematoma':ab,ti,kw OR 'subdural hematoma':ab,ti,kw OR 'bleeding, corpus callosum':ab,ti,kw OR 'brain bleeding':ab,ti,kw OR 'brain haemorrhage':ab,ti,kw OR 'brain haemorrhage, traumatic':ab,ti,kw OR 'brain hemorrhage, traumatic':ab,ti,kw OR 'brain microhaemorrhage':ab,ti,kw OR 'brain microhemorrhage':ab,ti,kw OR 'brain stem haemorrhage, traumatic':ab,ti,kw OR 'brain stem hemorrhage, traumatic':ab,ti,kw OR 'cerebral haemorrhage':ab,ti,kw OR 'cerebral haemorrhage, traumatic':ab,ti,kw OR 'cerebral hemorrhage':ab,ti,kw OR 'cerebral hemorrhage, traumatic':ab,ti,kw OR 'cerebral microbleed':ab,ti,kw OR 'corpus callosum bleeding':ab,ti,kw OR 'corpus callosum haemorrhage':ab,ti,kw OR 'corpus callosum hemorrhage':ab,ti,kw OR 'encephalorrhagia':ab,ti,kw OR 'haemorrhage, brain':ab,ti,kw OR 'haemorrhage, intracranial':ab,ti,kw OR 'haemorrhagic apoplexy':ab,ti,kw OR 'haemorrhagic stroke':ab,ti,kw OR 'haemorrhagic stroke intracerebral bleeding':ab,ti,kw OR 'hematencephalon':ab,ti,kw OR 'hemorrhage, brain':ab,ti,kw OR 'hemorrhage, intracranial':ab,ti,kw OR 'hemorrhagic apoplexy':ab,ti,kw OR 'hemorrhagic stroke':ab,ti,kw OR 'hemorrhagic stroke intracerebral bleeding':ab,ti,kw OR 'hypertensive intracranial haemorrhage':ab,ti,kw OR 'hypertensive intracranial hemorrhage':ab,ti,kw OR 'intracerebral bleeding':ab,ti,kw OR 'intracerebral haemorrhage':ab,ti,kw OR 'intracerebral hemorrhage':ab,ti,kw OR 'intracortical haemorrhage':ab,ti,kw OR 'intracortical hemorrhage':ab,ti,kw OR 'intracranial bleeding':ab,ti,kw OR 'intracranial haemorrhage':ab,ti,kw OR 'intracranial haemorrhage, hypertensive':ab,ti,kw OR 'intracranial haemorrhage, traumatic':ab,ti,kw OR 'intracranial haemorrhages':ab,ti,kw OR 'intracranial hemorrhage':ab,ti,kw OR 'intracranial hemorrhage, hypertensive':ab,ti,kw OR 'intracranial hemorrhage, traumatic':ab,ti,kw OR 'intracranial hemorrhages':ab,ti,kw OR 'intraventricular haemorrhage':ab,ti,kw OR 'intraventricular hemorrhage':ab,ti,kw OR 'periventricular haemorrhage':ab,ti,kw OR 'periventricular hemorrhage':ab,ti,kw OR 'posterior fossa haemorrhage':ab,ti,kw OR 'posterior fossa hemorrhage':ab,ti,kw OR 'traumatic brain haemorrhage':ab,ti,kw OR 'traumatic brain hemorrhage':ab,ti,kw OR 'traumatic brain stem haemorrhage':ab,ti,kw OR 'traumatic brain stem hemorrhage':ab,ti,kw OR 'traumatic cerebral haemorrhage':ab,ti,kw OR 'traumatic cerebral hemorrhage':ab,ti,kw OR 'traumatic intracranial haemorrhage':ab,ti,kw OR 'traumatic intracranial hemorrhage':ab,ti,kw OR 'brain hemorrhage':ab,ti,kw OR 'brain ventricle bleeding':ab,ti,kw OR 'brain ventricle haemorrhage':ab,ti,kw OR 'cerebral intraventricular hemorrhage':ab,ti,kw OR 'brain ventricle hemorrhage':ab,ti,kw OR 'cerebellar haemorrhage':ab,ti,kw OR 'cerebellar hemorrhage':ab,ti,kw OR 'cerebellum haemorrhage':ab,ti,kw OR 'haemorrhage, cerebellum':ab,ti,kw OR 'hemorrhage, cerebellum':ab,ti,kw OR 'cerebellum hemorrhage':ab,ti,kw OR 'massive cerebral bleeding':ab,ti,kw OR 'massive cerebral haemorrhage':ab,ti,kw OR 'massive cerebral hemorrhage':ab,ti,kw OR 'massive intracerebral bleeding':ab,ti,kw OR 'massive intracerebral haemorrhage':ab,ti,kw OR 'massive intracerebral hemorrhage':ab,ti,kw OR 'aneurysmal subarachnoid haemorrhage':ab,ti,kw OR 'aneurysmal subarachnoid hemorrhage':ab,ti,kw OR 'arachnoid haemorrhage, brain':ab,ti,kw OR 'arachnoid hemorrhage, brain':ab,ti,kw OR 'arachnoidal bleeding':ab,ti,kw OR 'arachnoidal haemorrhage':ab,ti,kw OR 'arachnoidal haemorrhage, brain':ab,ti,kw OR 'arachnoidal hemorrhage':ab,ti,kw OR 'arachnoidal hemorrhage, brain':ab,ti,kw OR 'bleeding, subarachnoid':ab,ti,kw OR 'brain arachnoid haemorrhage':ab,ti,kw OR 'brain arachnoid hemorrhage':ab,ti,kw OR 'haemorrhage, subarachnoid':ab,ti,kw OR 'hemorrhage, subarachnoid':ab,ti,kw OR 'spontaneous subarachnoid haemorrhage':ab,ti,kw OR 'spontaneous subarachnoid hemorrhage':ab,ti,kw OR 'subarachnoid bleeding':ab,ti,kw OR 'subarachnoid blood':ab,ti,kw OR 'subarachnoid haematoma':ab,ti,kw OR 'subarachnoid haemorrhage':ab,ti,kw OR 'subarachnoid haemorrhage, brain':ab,ti,kw OR 'subarachnoid haemorrhage, traumatic':ab,ti,kw OR 'subarachnoid hematoma':ab,ti,kw OR 'subarachnoid hemorrhage, brain':ab,ti,kw OR 'subarachnoid hemorrhage, traumatic':ab,ti,kw OR 'subarachnoid hemorrhagia':ab,ti,kw OR 'subarachnoidal bleeding':ab,ti,kw OR 'subarachnoidal haemorrhage':ab,ti,kw OR 'subarachnoidal hemorrhage':ab,ti,kw OR 'traumatic subarachnoid haemorrhage':ab,ti,kw OR 'traumatic subarachnoid hemorrhage':ab,ti,kw OR 'subarachnoid hemorrhage':ab,ti,kw OR 'brain cortex infarct':ab,ti,kw OR 'brain cortex infarction':ab,ti,kw OR 'brain infarct':ab,ti,kw OR 'cerebral cortex infarct':ab,ti,kw OR 'cerebral cortex infarction':ab,ti,kw OR 'cerebral infarct':ab,ti,kw OR 'cerebral infarction':ab,ti,kw OR 'cerebrovascular infarct':ab,ti,kw OR 'cerebrovascular infarction':ab,ti,kw OR 'cortical infarct':ab,ti,kw OR 'cortical infarction':ab,ti,kw OR 'hemisphere infarct':ab,ti,kw OR 'hemisphere infarction':ab,ti,kw OR 'hemispheric infarct':ab,ti,kw OR 'hemispheric infarction':ab,ti,kw OR 'infarction, brain':ab,ti,kw OR 'silent brain infarction':ab,ti,kw OR 'brain infarction':ab,ti,kw OR 'brain infarct size':ab,ti,kw OR 'cerebral infarct size':ab,ti,kw OR 'cerebral infarction size':ab,ti,kw OR 'cortical infarct size':ab,ti,kw OR 'cortical infarction size':ab,ti,kw OR 'infarct size, brain':ab,ti,kw OR 'brain infarction size':ab,ti,kw OR 'brain stem infarct':ab,ti,kw OR 'brain stem infarctions':ab,ti,kw OR 'brainstem infarct':ab,ti,kw OR 'brainstem infarction':ab,ti,kw OR 'infarction, brain stem':ab,ti,kw OR 'brain stem infarction':ab,ti,kw OR 'cadasil syndrome':ab,ti,kw OR 'cerebral autosomal dominant arteriopathy with subcortical infarct and leucoencephalopathy':ab,ti,kw OR 'cerebral autosomal dominant arteriopathy with subcortical infarct and leukoencephalopathy':ab,ti,kw OR 'cerebral autosomal dominant arteriopathy with subcortical infarcts and leucoencephalopathy':ab,ti,kw OR 'cerebral autosomal dominant arteriopathy with subcortical infarcts and leukoencephalopathy':ab,ti,kw OR 'cadasil':ab,ti,kw OR 'cerebellar infarct':ab,ti,kw OR 'cerebellar infarction':ab,ti,kw OR 'cerebellum infarct':ab,ti,kw OR 'cerebellum infarction':ab,ti,kw OR 'laci (lacunar infarction)':ab,ti,kw OR 'lacunar cerebral infarct':ab,ti,kw OR 'lacunar cerebral infarction':ab,ti,kw OR 'lacunar infarct':ab,ti,kw OR 'lacunar infarction':ab,ti,kw OR 'migraine infarct':ab,ti,kw OR 'migraine infarction':ab,ti,kw OR 'migrainous infarct':ab,ti,kw OR 'migrainous infarction':ab,ti,kw OR 'dementia, multi-infarct':ab,ti,kw OR 'dementia, multiinfarct':ab,ti,kw OR 'dementia, vascular':ab,ti,kw OR 'lacunar dementia':ab,ti,kw OR 'multi-infarct dementia':ab,ti,kw OR 'multi-infarction dementia':ab,ti,kw OR 'multiinfarction dementia':ab,ti,kw OR 'vascular dementia':ab,ti,kw OR 'multiinfarct dementia':ab,ti,kw OR 'acute brain ischaemia':ab,ti,kw OR 'acute brain ischemia':ab,ti,kw OR 'brain arterial insufficiency':ab,ti,kw OR 'brain circulation disorder':ab,ti,kw OR 'brain ischaemia':ab,ti,kw OR 'cerebral blood circulation disorder':ab,ti,kw OR 'cerebral blood flow disorder':ab,ti,kw OR 'cerebral circulation disorder':ab,ti,kw OR 'cerebral circulatory disorder':ab,ti,kw OR 'cerebral ischaemia':ab,ti,kw OR 'cerebral ischemia':ab,ti,kw OR 'cerebrovascular circulation disorder':ab,ti,kw OR 'cerebrovascular ischaemia':ab,ti,kw OR 'cerebrovascular ischemia':ab,ti,kw OR 'ischaemia cerebri':ab,ti,kw OR 'ischaemic brain disease':ab,ti,kw OR 'ischaemic encephalopathy':ab,ti,kw OR 'ischemia cerebri':ab,ti,kw OR 'ischemic brain disease':ab,ti,kw OR 'ischemic encephalopathy':ab,ti,kw OR 'neural ischaemia':ab,ti,kw OR 'neural ischemia':ab,ti,kw OR 'brain ischemia':ab,ti,kw OR 'anterior circulation ischemic event':ab,ti,kw OR 'anterior circulation ischemia':ab,ti,kw OR 'anterior circulation brain infarct':ab,ti,kw OR 'anterior circulation brain infarction':ab,ti,kw OR 'anterior circulation infarct':ab,ti,kw OR 'anterior circulation ischemic infarct':ab,ti,kw OR 'anterior circulation ischemic infarction':ab,ti,kw OR 'anterior circulatory infarct':ab,ti,kw OR 'anterior circulatory infarction':ab,ti,kw OR 'anterior circulation infarction':ab,ti,kw OR 'paci (partial anterior circulation infarct)':ab,ti,kw OR 'partial anterior circulation brain infarct':ab,ti,kw OR 'partial anterior circulation brain infarction':ab,ti,kw OR 'partial anterior circulation infarction':ab,ti,kw OR 'partial anterior circulatory infarct':ab,ti,kw OR 'partial anterior circulatory infarction':ab,ti,kw OR 'partial anterior circulation infarct':ab,ti,kw OR 'taci (total anterior circulation infarct)':ab,ti,kw OR 'total anterior circulation brain infarct':ab,ti,kw OR 'total anterior circulation brain infarction':ab,ti,kw OR 'total anterior circulation infarction':ab,ti,kw OR 'total anterior circulatory infarct':ab,ti,kw OR 'total anterior circulatory infarction':ab,ti,kw OR 'total anterior circulation infarct':ab,ti,kw OR 'anterior circulation ischaemic stroke':ab,ti,kw OR 'anterior circulation ischemic stroke':ab,ti,kw OR 'anterior circulation stroke syndrome':ab,ti,kw OR 'anterior circulatory stroke':ab,ti,kw OR 'anterior circulation stroke':ab,ti,kw OR 'posterior circulation ischemic event':ab,ti,kw OR 'posterior circulation ischemia':ab,ti,kw OR 'posterior circulation brain infarct':ab,ti,kw OR 'posterior circulation brain infarction':ab,ti,kw OR 'posterior circulation infarct':ab,ti,kw OR 'posterior circulation ischemic infarct':ab,ti,kw OR 'posterior circulation ischemic infarction':ab,ti,kw OR 'posterior circulatory infarct':ab,ti,kw OR 'posterior circulatory infarction':ab,ti,kw OR 'posterior circulation infarction':ab,ti,kw OR 'posterior circulation ischaemic stroke':ab,ti,kw OR 'posterior circulation ischemic stroke':ab,ti,kw OR 'posterior circulation stroke syndrome':ab,ti,kw OR 'posterior circulatory stroke':ab,ti,kw OR 'posterior circulation stroke':ab,ti,kw OR 'brain ischaemic attack':ab,ti,kw OR 'brain ischemic attack':ab,ti,kw OR 'brain transient ischaemic attack':ab,ti,kw OR 'brain transient ischemic attack':ab,ti,kw OR 'cerebral ischaemia, transient':ab,ti,kw OR 'cerebral ischemia, transient':ab,ti,kw OR 'circulatory epilepsy':ab,ti,kw OR 'epilepsy circulatory':ab,ti,kw OR 'ischaemic attack':ab,ti,kw OR 'ischaemic attack, transient':ab,ti,kw OR 'ischaemic cerebral attack':ab,ti,kw OR 'ischemic attack':ab,ti,kw OR 'ischemic attack, transient':ab,ti,kw OR 'ischemic cerebral attack':ab,ti,kw OR 'mini-stroke':ab,ti,kw OR 'tia':ab,ti,kw OR 'transient brain ischaemia':ab,ti,kw OR 'transient brain ischemia':ab,ti,kw OR 'transient cerebral ischaemia':ab,ti,kw OR 'transient cerebral ischemia':ab,ti,kw OR 'transient ischaemic attack':ab,ti,kw OR 'transient ischaemic seizure':ab,ti,kw OR 'transient ischemic seizure':ab,ti,kw OR 'transient ischemic attack':ab,ti,kw OR 'arteriosclerosis cerebri':ab,ti,kw OR 'atherosclerosis cerebri':ab,ti,kw OR 'atherosclerosis, brain':ab,ti,kw OR 'atherosclerotic cerebrovascular disease':ab,ti,kw OR 'brain arterio sclerosis':ab,ti,kw OR 'brain arteriosclerosis':ab,ti,kw OR 'brain atherosclerosis':ab,ti,kw OR 'cerebral arteriosclerosis':ab,ti,kw OR 'cerebral atherosclerotic disease':ab,ti,kw OR 'cerebral presclerosis':ab,ti,kw OR 'cerebrovascular arteriosclerosis':ab,ti,kw OR 'cerebrovascular atherosclerosis':ab,ti,kw OR 'cerebrovascular sclerosis':ab,ti,kw OR 'cerebrum atherosclerosis':ab,ti,kw OR 'intra-cranial atherosclerosis':ab,ti,kw OR 'intracranial arteriosclerosis':ab,ti,kw OR 'intracranial atherosclerosis':ab,ti,kw OR 'cerebral atherosclerosis':ab,ti,kw OR 'accident, cerebrovascular':ab,ti,kw OR 'acute cerebrovascular lesion':ab,ti,kw OR 'acute focal cerebral vasculopathy':ab,ti,kw OR 'acute stroke':ab,ti,kw OR 'apoplectic stroke':ab,ti,kw OR 'apoplexia':ab,ti,kw OR 'apoplexy':ab,ti,kw OR 'blood flow disturbance, brain':ab,ti,kw OR 'brain accident':ab,ti,kw OR 'brain attack':ab,ti,kw OR 'brain blood flow disturbance':ab,ti,kw OR 'brain insult':ab,ti,kw OR 'brain insultus':ab,ti,kw OR 'brain vascular accident':ab,ti,kw OR 'cerebral apoplexia':ab,ti,kw OR 'cerebral insult':ab,ti,kw OR 'cerebral stroke':ab,ti,kw OR 'cerebral vascular accident':ab,ti,kw OR 'cerebral vascular insufficiency':ab,ti,kw OR 'cerebrovascular arrest':ab,ti,kw OR 'cerebrovascular failure':ab,ti,kw OR 'cerebrovascular injury':ab,ti,kw OR 'cerebrovascular insufficiency':ab,ti,kw OR 'cerebrovascular insult':ab,ti,kw OR 'cerebrum vascular accident':ab,ti,kw OR 'cryptogenic stroke':ab,ti,kw OR 'cva':ab,ti,kw OR 'insultus cerebralis':ab,ti,kw OR 'ischaemic seizure':ab,ti,kw OR 'ischemic seizure':ab,ti,kw OR 'thrombotic stroke':ab,ti,kw OR 'cerebrovascular accident':ab,ti,kw OR 'brain stem stroke':ab,ti,kw OR 'brainstem stroke':ab,ti,kw OR 'embolic stroke':ab,ti,kw OR 'cardioembolic stroke':ab,ti,kw OR 'ischaemic stroke':ab,ti,kw OR 'ischemic stroke':ab,ti,kw OR 'lacs (lacunar syndrome)':ab,ti,kw OR 'lacunar cerebral stroke':ab,ti,kw OR 'lacunar stroke syndrome':ab,ti,kw OR 'lacunar syndrome':ab,ti,kw OR 'stroke, lacunar':ab,ti,kw OR 'lacunar stroke':ab,ti,kw OR 'brain blood vessel malformation':ab,ti,kw OR 'brain vascular anomaly':ab,ti,kw OR 'brain vascular malformation':ab,ti,kw OR 'cerebral vascular anomaly':ab,ti,kw OR 'cerebral vascular malformation':ab,ti,kw OR 'cerebrovascular anomaly':ab,ti,kw OR 'cerebrum vascular malformation':ab,ti,kw OR 'vascular malformation, cerebrum':ab,ti,kw OR 'arteriovenous aneurysm, brain':ab,ti,kw OR 'arteriovenous cerebral aneurysm':ab,ti,kw OR 'arteriovenous fistula, brain':ab,ti,kw OR 'brain aneurysm, arteriovenous':ab,ti,kw OR 'brain arteriovenous aneurysm':ab,ti,kw OR 'brain arteriovenous fistula':ab,ti,kw OR 'brain arteriovenous shunt':ab,ti,kw OR 'cerebral aneurysm, arteriovenous':ab,ti,kw OR 'cerebral arteriovenous fistula':ab,ti,kw OR 'cerebral arteriovenous malformation':ab,ti,kw OR 'cerebral arteriovenous malformations':ab,ti,kw OR 'intracranial arteriovenous malformations':ab,ti,kw OR 'brain arteriovenous malformation':ab,ti,kw OR 'meningeal angiomatosis':ab,ti,kw OR 'cerebral angiomatosis':ab,ti,kw OR 'leptomeningeal angiomatosis':ab,ti,kw OR 'angiomatosis cutanea cerebralis':ab,ti,kw OR 'encephalo trigeminal angiomatosis':ab,ti,kw OR 'encephalo-trigeminal angiomatosis':ab,ti,kw OR 'encephalofacial angiomatosis':ab,ti,kw OR 'encephalotrigeminal angiomatosis':ab,ti,kw OR 'krabbe weber dimitri disease':ab,ti,kw OR 'milles syndrome':ab,ti,kw OR 'schirmer syndrome':ab,ti,kw OR 'sturge weber disease':ab,ti,kw OR 'sturge weber krabbe syndrome':ab,ti,kw OR 'sturge-kalischer-weber syndrome':ab,ti,kw OR 'sturge-weber syndrome':ab,ti,kw OR 'sturge weber syndrome':ab,ti,kw OR 'aneurysm of the vein of galen':ab,ti,kw OR 'aneurysms of the vein of galen':ab,ti,kw OR 'galen vein aneurysm':ab,ti,kw OR 'great brain vein aneurysm':ab,ti,kw OR 'malformation of the vein of galen':ab,ti,kw OR 'malformations of the vein of galen':ab,ti,kw OR 'vein of galen aneurysm':ab,ti,kw OR 'vein of galen aneurysmal malformation':ab,ti,kw OR 'vein of galen aneurysmal malformations':ab,ti,kw OR 'vein of galen aneurysms':ab,ti,kw OR 'vein of galen arteriovenous malformation':ab,ti,kw OR 'vein of galen arteriovenous malformations':ab,ti,kw OR 'vein of galen malformations':ab,ti,kw OR 'vgam (vein of galen aneurysmal malformation)':ab,ti,kw OR 'vgm (vein of galen malformation)':ab,ti,kw OR 'vein of galen malformation':ab,ti,kw OR 'hypophyseal apoplexy':ab,ti,kw OR 'hypophysis haemorrhage':ab,ti,kw OR 'hypophysis hemorrhage':ab,ti,kw OR 'pituitary apoplexy':ab,ti,kw OR 'pituitary gland apoplexy':ab,ti,kw OR 'hypophysis apoplexy':ab,ti,kw OR 'lipohyalinitic degeneration':ab,ti,kw OR 'lipohyalinotic degeneration':ab,ti,kw OR 'lipohyalinotic lesion':ab,ti,kw OR 'lipohyalinotic occlusive disease':ab,ti,kw OR 'lipohyalinosis':ab,ti,kw OR 'melas':ab,ti,kw OR 'mitochondrial encephalomyopathy with lactic acidosis and stroke-like episodes':ab,ti,kw OR 'mitochondrial encephalomyopathy, lactic acidosis and stroke-like episodes':ab,ti,kw OR 'mitochondrial encephalomyopathy, lactic acidosis with stroke-like episodes':ab,ti,kw OR 'mitochondrial myopathy, encephalopathy, lactic acidosis and stroke-like episodes':ab,ti,kw OR 'myoencephalopathy, lactic acidosis, stroke':ab,ti,kw OR 'syndrome melas':ab,ti,kw OR 'melas syndrome':ab,ti,kw OR 'mitochondrial myopathy and sideroblastic anemia':ab,ti,kw OR 'mitochondrial myopathy, lactic acidosis and sideroblastic anemia':ab,ti,kw OR 'mlasa':ab,ti,kw OR 'myopathy, lactic acidosis and sideroblastic anemia':ab,ti,kw OR 'mlasa syndrome':ab,ti,kw OR 'brain vascular obstruction':ab,ti,kw OR 'cerebro-vascular occlusion':ab,ti,kw OR 'cerebro-vascular occlusive disease':ab,ti,kw OR 'cerebrovascular disease, occlusive':ab,ti,kw OR 'cerebrovascular obliteration':ab,ti,kw OR 'cerebrovascular obstruction':ab,ti,kw OR 'cerebrovascular occlusion':ab,ti,kw OR 'cerebrovascular occlusion disease':ab,ti,kw OR 'cerebrovascular occlusive disease':ab,ti,kw OR 'occlusive cerebro-vascular disease':ab,ti,kw OR 'occlusive cerebrovascular disease':ab,ti,kw OR 'brain phlebothrombosis':ab,ti,kw OR 'brain thromboembolism':ab,ti,kw OR 'brain thrombosis':ab,ti,kw OR 'cerebral thromboses':ab,ti,kw OR 'cerebro-vascular thrombosis':ab,ti,kw OR 'cerebrovascular thrombosis':ab,ti,kw OR 'intracranial thromboses':ab,ti,kw OR 'intracranial thrombosis':ab,ti,kw OR 'thromboembolism, brain':ab,ti,kw OR 'thrombosis cerebri':ab,ti,kw OR 'thrombosis, intracranial':ab,ti,kw OR 'cerebral thrombosis':ab,ti,kw OR 'cavernous sinus thrombosis syndrome':ab,ti,kw OR 'cavernous thrombosis':ab,ti,kw OR 'foix syndrome':ab,ti,kw OR 'sinus cavernosus thrombosis':ab,ti,kw OR 'thrombosis, cavernous sinus':ab,ti,kw OR 'thrombosis, sinus cavernosus':ab,ti,kw OR 'cavernous sinus thrombosis':ab,ti,kw OR 'cavernous sinus thrombo-phlebitis':ab,ti,kw OR 'cavernous thrombophlebitis':ab,ti,kw OR 'thrombophlebitis of the cavernous sinus':ab,ti,kw OR 'cavernous sinus thrombophlebitis':ab,ti,kw OR 'thrombosis, lateral sinus':ab,ti,kw OR 'transverse sinus thrombosis':ab,ti,kw OR 'lateral sinus thrombosis':ab,ti,kw OR 'longitudinal sinus thrombosis':ab,ti,kw OR 'sinus sagittalis thrombosis':ab,ti,kw OR 'superior longitudinal sinus thrombosis':ab,ti,kw OR 'superior sagittal sinus thrombosis':ab,ti,kw OR 'thrombosis, sagittal sinus':ab,ti,kw OR 'sagittal sinus thrombosis':ab,ti,kw OR 'syndrome, sneddon':ab,ti,kw OR 'sneddon syndrome':ab,ti,kw OR 'posterior encephalopathy':ab,ti,kw OR 'posterior leucoencephalopathy syndrome':ab,ti,kw OR 'posterior leukoencephalopathy':ab,ti,kw OR 'posterior leukoencephalopathy syndrome':ab,ti,kw OR 'posterior reversible encephalopathy':ab,ti,kw OR 'posterior reversible leucoencephalopathy':ab,ti,kw OR 'posterior reversible leucoencephalopathy syndrome':ab,ti,kw OR 'posterior reversible leukoencephalopathy':ab,ti,kw OR 'posterior reversible leukoencephalopathy syndrome':ab,ti,kw OR 'pres (posterior reversible encephalopathy syndrome)':ab,ti,kw OR 'reversible posterior leucoencephalopathy':ab,ti,kw OR 'reversible posterior leucoencephalopathy syndrome':ab,ti,kw OR 'reversible posterior leukoencephalopathy':ab,ti,kw OR 'reversible posterior leukoencephalopathy syndrome':ab,ti,kw OR 'rpls (reversible posterior leucoencephalopathy syndrome)':ab,ti,kw OR 'rpls (reversible posterior leukoencephalopathy syndrome)':ab,ti,kw OR 'posterior reversible encephalopathy syndrome':ab,ti,kw OR 'subcortical ischaemic depression':ab,ti,kw OR 'subcortical ischemic depression':ab,ti,kw OR 'vascular depression':ab,ti,kw OR 'arterial vertebrobasillaris insufficiency':ab,ti,kw OR 'brachial basilar insufficiency':ab,ti,kw OR 'vertebral basilar insufficiency':ab,ti,kw OR 'vertebro basilar insufficiency':ab,ti,kw OR 'vertebrobasilar disease':ab,ti,kw OR 'vertebrobasilar ischaemia':ab,ti,kw OR 'vertebrobasilar ischemia':ab,ti,kw OR 'vertebrobasilar syndrome':ab,ti,kw OR 'vertebrobasilar insufficiency':ab,ti,kw	473723
#3	#1 OR #2	665669
#4	'pulse wave velocity'/exp	6779
#5	'cardiac-ankle vascular index':ab,ti,kw OR 'cardio ankle vascular index':ab,ti,kw OR 'cardio-vascular ankle index':ab,ti,kw OR 'cardioankle vascular index':ab,ti,kw OR 'cardiovascular ankle index':ab,ti,kw OR 'cavi (cardio-ankle vascular index)':ab,ti,kw OR 'cardio-ankle vascular index':ab,ti,kw OR 'pwv (pulse wave velocity)':ab,ti,kw OR 'aorta pulse wave velocity':ab,ti,kw OR 'aortal pulse wave velocity':ab,ti,kw OR 'aortic pwv':ab,ti,kw OR 'pulse wave velocity of aorta':ab,ti,kw OR 'pulse wave velocity of the aorta':ab,ti,kw OR 'aortic pulse wave velocity':ab,ti,kw OR 'arterial pulse velocity':ab,ti,kw OR 'arterial pwv':ab,ti,kw OR 'arterial wave velocity':ab,ti,kw OR 'artery pulse velocity':ab,ti,kw OR 'artery pulse wave velocity':ab,ti,kw OR 'arterial pulse wave velocity':ab,ti,kw OR 'ankle-brachial pulse wave velocity':ab,ti,kw OR 'bapwv (brachial-ankle pulse wave velocity)':ab,ti,kw OR 'brachial-ankle pulse velocity':ab,ti,kw OR 'brachial-ankle wave velocity':ab,ti,kw OR 'brachial-ankle pulse wave velocity':ab,ti,kw OR 'carotid-femoral apwv':ab,ti,kw OR 'carotid-femoral wave velocity':ab,ti,kw OR 'cf-pwv (carotid-femoral pulse wave velocity)':ab,ti,kw OR 'femoral carotid pulse velocity':ab,ti,kw OR 'femoral-carotid pulse wave velocity':ab,ti,kw OR 'carotid-femoral pulse wave velocity':ab,ti,kw OR 'carotid-radial pulse wave velocity (pwv-cr)':ab,ti,kw OR 'pwv-cr (carotid-radial pulse wave velocity)':ab,ti,kw OR 'carotid-radial pulse wave velocity':ab,ti,kw	12121
#6	#4 OR #5	15850
#7	#3 AND #6	684

Ovid MEDLINE

**Table 3:** Search strings used in Ovid MEDLINE

Search strings used in Ovid MEDLINE		
#1	exp Pulse Wave Analysis/	7069
#2	('cardiac-ankle vascular index' or 'cardio ankle vascular index' or 'cardio-vascular ankle index' or 'cardioankle vascular index' or 'cardiovascular ankle index' or 'cardio-ankle vascular index' or 'cardio-ankle vascular index' or 'pwv' or 'pulse wave velocity' or 'pulse wave velocity of aorta' or 'pulse wave velocity of the aorta' or 'carotid-femoral apwv' or 'carotid-femoral wave velocity' or 'cf-pwv' or 'femoral carotid pulse velocity' or 'carotid-radial pulse wave velocity' or 'carotid-radial pulse wave velocity' or 'carotid-radial pulse wave velocity').ab,ti,kf.	13374
#3	#1 OR #2	14803
#4	exp Basal Ganglia Cerebrovascular Disease/ or exp Brain Ischemia/ or exp Cerebral Small Vessel Diseases/ or exp Dementia, Vascular/ or exp Intracranial Arteriosclerosis/ or exp Intracranial Arteriovenous Malformations/ or exp "Intracranial Embolism and Thrombosis"/ or exp Intracranial Hemorrhages/ or exp Sneddon Syndrome/ or exp Stroke/ or exp Vascular Depression/ or exp Vascular Headaches/ or exp Vasospasm, Intracranial/	345443
#5	('brain angiopathy' or 'brain circulation failure' or 'brain vascular disease' or 'brain vasculopathy' or 'cerebral angiopathy' or 'cerebral small vessel disease' or 'cerebral small vessel diseases' or 'cerebral vascular disease' or 'cerebral vascular disorder' or 'cerebral vascular disturbance' or 'cerebral vascular lesion' or 'cerebral vasculopathy' or 'cerebroangiopathy' or 'cerebrovascular damage' or 'cerebrovascular disorder' or 'cerebrovascular disturbance' or 'cerebrovascular lesion' or 'cerebrovascular pathology' or 'cerebrovascular syndrome' or 'cerebrovasculopathy' or 'cerebrovascular disease' or 'basal ganglia cerebrovascular disease' or 'basal ganglia haemorrhage' or 'basal ganglia hemorrhage' or 'basal ganglion cerebrovascular disease' or 'basal ganglion haemorrhage' or 'cerebrovascular disease, basal ganglion' or 'haemorrhage, basal ganglion' or 'hemorrhage, basal ganglion' or 'basal ganglion hemorrhage' or 'haemorrhage, putamen' or 'hemorrhage, putamen' or 'putamen haematoma' or 'putamen haemorrhage' or 'putamen hematoma' or 'putamen hemorrhage' or 'putaminal haematoma' or 'putaminal haemorrhage' or 'putaminal hematoma' or 'putaminal hemorrhage' or 'brain haematoma' or 'cerebral haematoma' or 'cerebral hematoma' or 'haematoma, brain' or 'hematoma, brain' or 'interhemispheric haematoma' or 'interhemispheric hematoma' or 'intracerebral haematoma' or 'intracerebral hematoma' or 'intracranial haematoma' or 'intracranial hematoma' or 'brain hematoma' or 'acute subdural haematoma' or 'acute subdural hematoma' or 'chronic subdural haematoma' or 'chronic subdural haematomata' or 'chronic subdural hematoma' or 'chronic subdural hematomata' or 'haemorrhagic pachymeningitis' or 'hemorrhagic pachymeningitis' or 'intracranial subdural haematoma' or 'intracranial subdural hematoma' or 'subdural bleeding' or 'subdural haematoma' or 'subdural haemorrhage' or 'subdural hemorrhage' or 'subepidural haematoma' or 'subepidural hematoma' or 'subdural hematoma' or 'brain bleeding' or 'brain haemorrhage' or 'brain hemorrhage, traumatic' or 'brain microhaemorrhage' or 'brain microhemorrhage' or 'brain stem haemorrhage, traumatic' or 'brain stem hemorrhage, traumatic' or 'cerebral haemorrhage' or 'cerebral hemorrhage' or 'cerebral microbleed' or 'corpus callosum bleeding' or 'corpus callosum haemorrhage' or 'corpus callosum hemorrhage' or 'encephalorrhagia' or 'haemorrhagic apoplexy' or 'haemorrhagic stroke' or 'haemorrhagic stroke intracerebral bleeding' or 'hematencephalon' or 'hemorrhage, brain' or 'hemorrhage, intracranial' or 'hemorrhagic apoplexy' or 'hemorrhagic stroke' or 'hemorrhagic stroke intracerebral bleeding' or 'hypertensive intracranial haemorrhage' or 'hypertensive intracranial hemorrhage' or 'intracerebral bleeding' or 'intracerebral haemorrhage' or 'intracerebral hemorrhage' or 'intracortical haemorrhage' or 'intracortical hemorrhage' or 'intracranial bleeding' or 'intracranial haemorrhage' or 'intracranial haemorrhage, hypertensive' or 'intracranial haemorrhage, traumatic' or 'intracranial haemorrhages' or 'intracranial hemorrhage' or 'intracranial hemorrhage, hypertensive' or 'intracranial hemorrhage, traumatic' or 'intracranial hemorrhages' or 'intraventricular haemorrhage' or 'intraventricular hemorrhage' or 'periventricular haemorrhage' or 'periventricular hemorrhage' or 'posterior fossa haemorrhage' or 'posterior fossa hemorrhage' or 'traumatic brain haemorrhage' or 'traumatic brain hemorrhage' or 'traumatic brain stem haemorrhage' or 'traumatic brain stem hemorrhage' or 'traumatic cerebral haemorrhage' or 'traumatic cerebral hemorrhage' or 'traumatic intracranial haemorrhage' or 'traumatic intracranial hemorrhage' or 'brain hemorrhage' or 'brain ventricle bleeding' or 'brain ventricle haemorrhage' or 'cerebral intraventricular hemorrhage' or 'brain ventricle hemorrhage' or 'cerebellar haemorrhage' or 'cerebellar hemorrhage' or 'cerebellum haemorrhage' or 'haemorrhage, cerebellum' or 'hemorrhage, cerebellum' or 'cerebellum hemorrhage' or 'massive cerebral bleeding' or 'massive cerebral haemorrhage' or 'massive cerebral hemorrhage' or 'massive intracerebral bleeding' or 'massive intracerebral haemorrhage' or 'massive intracerebral hemorrhage' or 'aneurysmal subarachnoid haemorrhage' or 'aneurysmal subarachnoid hemorrhage' or 'arachnoid haemorrhage, brain' or 'arachnoid hemorrhage, brain' or 'arachnoidal bleeding' or 'arachnoidal haemorrhage' or 'arachnoidal haemorrhage, brain' or 'arachnoidal hemorrhage' or 'arachnoidal hemorrhage, brain' or 'bleeding, subarachnoid' or 'brain arachnoid haemorrhage' or 'brain arachnoid hemorrhage' or 'haemorrhage, subarachnoid' or 'hemorrhage, subarachnoid' or 'spontaneous subarachnoid haemorrhage' or 'spontaneous subarachnoid hemorrhage' or 'subarachnoid bleeding' or 'subarachnoid blood' or 'subarachnoid haematoma' or 'subarachnoid haemorrhage' or 'subarachnoid haemorrhage, brain' or 'subarachnoid haemorrhage, traumatic' or 'subarachnoid hematoma' or 'subarachnoid hemorrhage, brain' or 'subarachnoid hemorrhage, traumatic' or 'subarachnoid hemorrhagia' or 'subarachnoidal bleeding' or 'subarachnoidal haemorrhage' or 'subarachnoidal hemorrhage' or 'traumatic subarachnoid haemorrhage' or 'traumatic subarachnoid hemorrhage' or 'subarachnoid hemorrhage' or 'brain cortex infarct' or 'brain cortex infarction' or 'brain infarct' or 'cerebral cortex infarct' or 'cerebral cortex infarction' or 'cerebral infarct' or 'cerebral infarction' or 'cerebrovascular infarct' or 'cerebrovascular infarction' or 'cortical infarct' or 'cortical infarction' or 'hemisphere infarct' or 'hemisphere infarction' or 'hemispheric infarct' or 'hemispheric infarction' or 'infarction, brain' or 'silent brain infarction' or 'brain infarction' or 'brain infarct size' or 'cerebral infarct size' or 'cerebral infarction size' or 'cortical infarct size' or 'cortical infarction size' or 'infarct size, brain' or 'brain infarction size' or 'brain stem infarct' or 'brain stem infarctions' or 'brainstem infarct' or 'brainstem infarction' or 'brain stem infarction' or 'cadasil syndrome' or 'cadasil' or 'cerebellar infarct' or 'cerebellar infarction' or 'cerebellum infarct' or 'cerebellum infarction' or 'lacunar infarction' or 'lacunar cerebral infarct' or 'lacunar cerebral infarction' or 'lacunar infarct' or 'lacunar infarction' or 'migraine infarct' or 'migraine infarction' or 'migrainous infarct' or 'migrainous infarction' or 'vascular dementia' or 'lacunar dementia' or 'multi-infarct dementia' or 'multi-infarction dementia' or 'vascular dementia' or 'acute brain ischaemia' or 'acute brain ischemia' or 'brain arterial insufficiency' or 'brain circulation disorder' or 'brain ischaemia' or 'cerebral blood circulation disorder' or 'cerebral blood flow disorder' or 'cerebral circulation disorder' or 'cerebral circulatory disorder' or 'cerebral ischaemia' or 'cerebral ischemia' or 'cerebrovascular circulation disorder' or 'cerebrovascular ischaemia' or 'cerebrovascular ischemia' or 'ischaemia cerebri' or 'ischaemic brain disease' or 'ischaemic encephalopathy' or 'ischemia cerebri' or 'ischemic brain disease' or 'ischemic encephalopathy' or 'neural ischaemia' or 'neural ischemia' or 'brain ischemia' or 'anterior circulation ischemic event' or 'anterior circulation ischemia' or 'anterior circulation brain infarct' or 'anterior circulation brain infarction' or 'anterior circulation infarct' or 'anterior circulation ischemic infarct' or 'anterior circulation ischemic infarction' or 'anterior circulatory infarct' or 'anterior circulatory infarction' or 'anterior circulation infarction' or 'partial anterior circulation infarct' or 'partial anterior circulation brain infarct' or 'partial anterior circulation brain infarction' or 'partial anterior circulation infarction' or 'partial anterior circulatory infarct' or 'partial anterior circulatory infarction' or 'partial anterior circulation infarct' or 'total anterior circulation infarct' or 'total anterior circulation brain infarction' or 'total anterior circulation infarction' or 'anterior circulation ischaemic stroke' or 'anterior circulation ischemic stroke' or 'anterior circulation stroke syndrome' or 'anterior circulatory stroke' or 'anterior circulation stroke' or 'posterior circulation ischemic event' or 'posterior circulation ischemia' or 'posterior circulation brain infarct' or 'posterior circulation brain infarction' or 'posterior circulation infarct' or 'posterior circulation ischemic infarct' or 'posterior circulation ischemic infarction' or 'posterior circulatory infarct' or 'posterior circulatory infarction' or 'posterior circulation infarction' or 'posterior circulation ischaemic stroke' or 'posterior circulation ischemic stroke' or 'posterior circulation stroke syndrome' or 'posterior circulatory stroke' or 'posterior circulation stroke' or 'brain ischaemic attack' or 'brain ischemic attack' or 'brain transient ischaemic attack' or 'brain transient ischemic attack' or 'cerebral ischaemia, transient' or 'cerebral ischemia, transient' or 'circulatory epilepsy' or 'epilepsy circulatory' or 'ischaemic attack' or 'ischaemic attack, transient' or 'ischaemic cerebral attack' or 'ischemic attack' or 'ischemic cerebral attack' or 'mini-stroke' or 'transient brain ischaemia' or 'transient brain ischemia' or 'transient cerebral ischaemia' or 'transient cerebral ischemia' or 'transient ischaemic attack' or 'transient ischaemic seizure' or 'transient ischemic seizure' or 'transient ischemic attack' or 'arteriosclerosis cerebri' or 'atherosclerosis cerebri' or 'atherosclerotic cerebrovascular disease' or 'brain arteriosclerosis' or 'brain atherosclerosis' or 'cerebral arteriosclerosis' or 'cerebral atherosclerotic disease' or 'cerebral presclerosis' or 'cerebrovascular arteriosclerosis' or 'cerebrovascular atherosclerosis' or 'cerebrovascular sclerosis' or 'cerebrum atherosclerosis' or 'intracranial arteriosclerosis' or 'intracranial atherosclerosis' or 'cerebral atherosclerosis' or 'acute cerebrovascular lesion' or 'acute focal cerebral vasculopathy' or 'acute stroke' or 'apoplectic stroke' or 'apoplexia' or 'apoplexy' or 'brain accident' or 'brain attack' or 'brain blood flow disturbance' or 'brain insult' or 'brain insultus' or 'brain vascular accident' or 'cerebral apoplexia' or 'cerebral insult' or 'cerebral stroke' or 'cerebral vascular accident' or 'cerebral vascular insufficiency' or 'cerebrovascular arrest' or 'cerebrovascular failure' or 'cerebrovascular injury' or 'cerebrovascular insufficiency' or 'cerebrovascular insult' or 'cerebrum vascular accident' or 'cryptogenic stroke' or 'insultus cerebralis' or 'ischaemic seizure' or 'ischemic seizure' or 'thrombotic stroke' or 'cerebrovascular accident' or 'brain stem stroke' or 'brainstem stroke' or 'embolic stroke' or 'cardioembolic stroke' or 'ischaemic stroke' or 'ischemic stroke' or 'lacunar syndrome' or 'lacunar cerebral stroke' or 'lacunar stroke syndrome' or 'lacunar syndrome' or 'lacunar stroke' or 'brain blood vessel malformation' or 'brain vascular anomaly' or 'brain vascular malformation' or 'cerebral vascular anomaly' or 'cerebral vascular malformation' or 'cerebrovascular anomaly' or 'cerebrum vascular malformation' or 'arteriovenous cerebral aneurysm' or 'brain arteriovenous aneurysm' or 'brain arteriovenous fistula' or 'brain arteriovenous shunt' or 'cerebral arteriovenous fistula' or 'cerebral arteriovenous malformation' or 'cerebral arteriovenous malformations' or 'intracranial arteriovenous malformations' or 'brain arteriovenous malformation' or 'meningeal angiomatosis' or 'cerebral angiomatosis' or 'leptomeningeal angiomatosis' or 'angiomatosis cutanea cerebralis' or 'encephalo trigeminal angiomatosis' or 'encephalofacial angiomatosis' or 'encephalotrigeminal angiomatosis' or 'krabbe weber dimitri disease' or 'milles syndrome' or 'schirmer syndrome' or 'sturge weber disease' or 'sturge weber krabbe syndrome' or 'sturge-kalischer-weber syndrome' or 'sturge-weber syndrome' or 'sturge weber syndrome' or 'aneurysm of the vein of galen' or 'aneurysms of the vein of galen' or 'galen vein aneurysm' or 'great brain vein aneurysm' or 'vein of galen aneurysm' or 'vein of galen aneurysmal malformation' or 'vein of galen aneurysm' or 'vein of galen arteriovenous malformation' or 'vein of galen malformation' or 'hypophyseal apoplexy' or 'hypophysis haemorrhage' or 'hypophysis hemorrhage' or 'pituitary apoplexy' or 'pituitary gland apoplexy' or 'hypophysis apoplexy' or 'lipohyalinitic degeneration' or 'lipohyalinotic degeneration' or 'lipohyalinotic lesion' or 'lipohyalinotic occlusive disease' or 'lipohyalinosis' or 'melas' or 'mitochondrial encephalomyopathy with lactic acidosis) and stroke-like episodes') or 'melas syndrome' or 'brain vascular obstruction' or 'cerebrovascular disease, occlusive' or 'cerebrovascular obliteration' or 'cerebrovascular obstruction' or 'cerebrovascular occlusion' or 'cerebrovascular occlusion disease' or 'cerebrovascular occlusive disease' or 'occlusive cerebrovascular disease' or 'brain phlebothrombosis' or 'brain thromboembolism' or 'brain thrombosis' or 'cerebral thromboses' or 'cerebrovascular thrombosis' or 'intracranial thromboses' or 'intracranial thrombosis' or 'thrombosis cerebri' or 'cerebral thrombosis' or 'cavernous sinus thrombosis syndrome' or 'cavernous thrombosis' or 'foix syndrome' or 'cavernous sinus thrombosis' or 'cavernous thrombophlebitis' or 'cavernous sinus thrombophlebitis' or 'transverse sinus thrombosis' or 'lateral sinus thrombosis' or 'longitudinal sinus thrombosis' or 'sinus sagittalis thrombosis' or 'superior longitudinal sinus thrombosis' or 'superior sagittal sinus thrombosis' or 'sagittal sinus thrombosis' or 'sneddon syndrome' or 'posterior encephalopathy' or 'posterior leucoencephalopathy syndrome' or 'posterior leukoencephalopathy' or 'posterior leukoencephalopathy syndrome' or 'posterior reversible encephalopathy' or 'posterior reversible leucoencephalopathy' or 'posterior reversible leucoencephalopathy syndrome' or 'posterior reversible leukoencephalopathy' or 'posterior reversible leukoencephalopathy syndrome' or 'posterior reversible encephalopathy syndrome' or 'reversible posterior leucoencephalopathy' or 'reversible posterior leucoencephalopathy syndrome' or 'reversible posterior leukoencephalopathy' or 'reversible posterior leukoencephalopathy syndrome' or 'reversible posterior leucoencephalopathy syndrome' or 'reversible posterior leukoencephalopathy syndrome' or 'posterior reversible encephalopathy syndrome' or 'subcortical ischaemic depression' or 'subcortical ischemic depression' or 'vascular depression' or 'arterial vertebrobasillaris insufficiency' or 'brachial basilar insufficiency' or 'vertebral basilar insufficiency' or 'vertebrobasilar insufficiency' or 'vertebrobasilar disease' or 'vertebrobasilar ischaemia' or 'vertebrobasilar ischemia' or 'vertebrobasilar syndrome').ab,ti,kf.	9907
#6	#4 OR #5	351124
#7	#3 AND #6	321

Web of Science

**Table 4:** Search strings used in Web of Science

Search strings used in Web of Science		
#1	“cardiac-ankle vascular index” OR “cardio ankle vascular index” OR “cardio-vascular ankle index” OR “cardioankle vascular index” OR “cardiovascular ankle index” OR “cavi (cardio-ankle vascular index)” OR “cardio-ankle vascular index” OR “pwv (pulse wave velocity)” OR “aorta pulse wave velocity” OR “aortal pulse wave velocity” OR “aortic pwv” OR “pulse wave velocity of aorta” OR “pulse wave velocity of the aorta” OR “aortic pulse wave velocity” OR “arterial pulse velocity” OR “arterial pwv” OR “arterial wave velocity” OR “artery pulse velocity” OR “artery pulse wave velocity” OR “arterial pulse wave velocity” OR “ankle-brachial pulse wave velocity” OR “bapwv (brachial-ankle pulse wave velocity)” OR “brachial-ankle pulse velocity” OR “brachial-ankle wave velocity” OR “brachial-ankle pulse wave velocity” OR “carotid-femoral apwv” OR “carotid-femoral wave velocity” OR “cf-pwv (carotid-femoral pulse wave velocity)” OR “femoral carotid pulse velocity” OR “femoral-carotid pulse wave velocity” OR “carotid-femoral pulse wave velocity” OR “carotid-radial pulse wave velocity (pwv-cr)” OR “pwv-cr (carotid-radial pulse wave velocity)” OR “carotid-radial pulse wave velocity” (Topic)	7113
#2	“brain angiopathy” OR “brain circulation failure” OR “brain vascular disease” OR “brain vasculopathy” OR “cerebral angiopathy” OR “cerebral small vessel disease” OR “cerebral small vessel diseases” OR “cerebral vascular disease” OR “cerebral vascular disorder” OR “cerebral vascular disturbance” OR “cerebral vascular lesion” OR “cerebral vasculopathy” OR “cerebro-vascular damage” OR “cerebro-vascular disease” OR “cerebro-vascular disorder” OR “cerebro-vascular disturbance” OR “cerebro-vascular lesion” OR “cerebro-vascular pathology” OR “cerebro-vascular syndrome” OR “cerebro-vasculopathy” OR “cerebroangiopathy” OR “cerebrovascular damage” OR “cerebrovascular disorder” OR “cerebrovascular disorders” OR “cerebrovascular disturbance” OR “cerebrovascular lesion” OR “cerebrovascular pathology” OR “cerebrovascular syndrome” OR “cerebrovasculopathy” OR “cerebrovascular disease” OR “basal ganglia cerebrovascular disease” OR “basal ganglia haemorrhage” OR “basal ganglia hemorrhage” OR “basal ganglion cerebrovascular disease” OR “basal ganglion haemorrhage” OR “cerebrovascular disease, basal ganglion” OR “haemorrhage, basal ganglion” OR “hemorrhage, basal ganglion” OR “basal ganglion hemorrhage” OR “haemorrhage, putamen” OR “hemorrhage, putamen” OR “putamen haematoma” OR “putamen haemorrhage” OR “putamen hematoma” OR “putamen hemorrhage” OR “putaminal haematoma” OR “putaminal haemorrhage” OR “putaminal hematoma” OR “putaminal hemorrhage” OR “brain haematoma” OR “cerebral haematoma” OR “cerebral hematoma” OR “haematoma, brain” OR “hematoma, brain” OR “interhemispheric haematoma” OR “interhemispheric hematoma” OR “intracerebral haematoma” OR “intracerebral hematoma” OR “intracranial haematoma” OR “intracranial hematoma” OR “brain hematoma” OR “acute subdural haematoma” OR “acute subdural hematoma” OR “chronic subdural haematoma” OR “chronic subdural haematomata” OR “chronic subdural hematoma” OR “chronic subdural hematomata” OR “haematoma, subdural” OR “haematoma, subdural, acute” OR “haematoma, subdural, chronic” OR “haematoma, subdural, intracranial” OR “haemorrhage, subdural” OR “haemorrhagic pachymeningitis” OR “hematoma, subdural” OR “hematoma, subdural, acute” OR “hematoma, subdural, chronic” OR “hematoma, subdural, intracranial” OR “hemorrhage, subdural” OR “hemorrhagic pachymeningitis” OR “intracranial subdural haematoma” OR “intracranial subdural haematomas” OR “intracranial subdural haematomata” OR “intracranial subdural hematoma” OR “intracranial subdural hematomas” OR “intracranial subdural hematomata” OR “pachymeningiosis haemorrhagica interna” OR “pachymeningitis haemorrhagica” OR “subdural bleeding” OR “subdural haematoma” OR “subdural haemorrhage” OR “subdural hemorrhage” OR “subepidural haematoma” OR “subepidural hematoma” OR “subdural hematoma” OR “bleeding, corpus callosum” OR “brain bleeding” OR “brain haemorrhage” OR “brain haemorrhage, traumatic” OR “brain hemorrhage, traumatic” OR “brain microhaemorrhage” OR “brain microhemorrhage” OR “brain stem haemorrhage, traumatic” OR “brain stem hemorrhage, traumatic” OR “cerebral haemorrhage” OR “cerebral haemorrhage, traumatic” OR “cerebral hemorrhage” OR “cerebral hemorrhage, traumatic” OR “cerebral microbleed” OR “corpus callosum bleeding” OR “corpus callosum haemorrhage” OR “corpus callosum hemorrhage” OR “encephalorrhagia” OR “haemorrhage, brain” OR “haemorrhage, intracranial” OR “haemorrhagic apoplexy” OR “haemorrhagic stroke” OR “haemorrhagic stroke intracerebral bleeding” OR “hematencephalon” OR “hemorrhage, brain” OR “hemorrhage, intracranial” OR “hemorrhagic apoplexy” OR “hemorrhagic stroke” OR “hemorrhagic stroke intracerebral bleeding” OR “hypertensive intracranial haemorrhage” OR “hypertensive intracranial hemorrhage” OR “intracerebral bleeding” OR “intracerebral haemorrhage” OR “intracerebral hemorrhage” OR “intracortical haemorrhage” OR “intracortical hemorrhage” OR “intracranial bleeding” OR “intracranial haemorrhage” OR “intracranial haemorrhage, hypertensive” OR “intracranial haemorrhage, traumatic” OR “intracranial haemorrhages” OR “intracranial hemorrhage” OR “intracranial hemorrhage, hypertensive” OR “intracranial hemorrhage, traumatic” OR “intracranial hemorrhages” OR “intraventricular haemorrhage” OR “intraventricular hemorrhage” OR “periventricular haemorrhage” OR “periventricular hemorrhage” OR “posterior fossa haemorrhage” OR “posterior fossa hemorrhage” OR “traumatic brain haemorrhage” OR “traumatic brain hemorrhage” OR “traumatic brain stem haemorrhage” OR “traumatic brain stem hemorrhage” OR “traumatic cerebral haemorrhage” OR “traumatic cerebral hemorrhage” OR “traumatic intracranial haemorrhage” OR “traumatic intracranial hemorrhage” OR “brain hemorrhage” OR “brain ventricle bleeding” OR “brain ventricle haemorrhage” OR “cerebral intraventricular hemorrhage” OR “brain ventricle hemorrhage” OR “cerebellar haemorrhage” OR “cerebellar hemorrhage” OR “cerebellum haemorrhage” OR “haemorrhage, cerebellum” OR “hemorrhage, cerebellum” OR “cerebellum hemorrhage” OR “massive cerebral bleeding” OR “massive cerebral haemorrhage” OR “massive cerebral hemorrhage” OR “massive intracerebral bleeding” OR “massive intracerebral haemorrhage” OR “massive intracerebral hemorrhage” OR “aneurysmal subarachnoid haemorrhage” OR “aneurysmal subarachnoid hemorrhage” OR “arachnoid haemorrhage, brain” OR “arachnoid hemorrhage, brain” OR “arachnoidal bleeding” OR “arachnoidal haemorrhage” OR “arachnoidal haemorrhage, brain” OR “arachnoidal hemorrhage” OR “arachnoidal hemorrhage, brain” OR “bleeding, subarachnoid” OR “brain arachnoid haemorrhage” OR “brain arachnoid hemorrhage” OR “haemorrhage, subarachnoid” OR “hemorrhage, subarachnoid” OR “spontaneous subarachnoid haemorrhage” OR “spontaneous subarachnoid hemorrhage” OR “subarachnoid bleeding” OR “subarachnoid blood” OR “subarachnoid haematoma” OR “subarachnoid haemorrhage” OR “subarachnoid haemorrhage, brain” OR “subarachnoid haemorrhage, traumatic” OR “subarachnoid hematoma” OR “subarachnoid hemorrhage, brain” OR “subarachnoid hemorrhage, traumatic” OR “subarachnoid hemorrhagia” OR “subarachnoidal bleeding” OR “subarachnoidal haemorrhage” OR “subarachnoidal hemorrhage” OR “traumatic subarachnoid haemorrhage” OR “traumatic subarachnoid hemorrhage” OR “subarachnoid hemorrhage” OR “brain cortex infarct” OR “brain cortex infarction” OR “brain infarct” OR “cerebral cortex infarct” OR “cerebral cortex infarction” OR “cerebral infarct” OR “cerebral infarction” OR “cerebrovascular infarct” OR “cerebrovascular infarction” OR “cortical infarct” OR “cortical infarction” OR “hemisphere infarct” OR “hemisphere infarction” OR “hemispheric infarct” OR “hemispheric infarction” OR “infarction, brain” OR “silent brain infarction” OR “brain infarction” OR “brain infarct size” OR “cerebral infarct size” OR “cerebral infarction size” OR “cortical infarct size” OR “cortical infarction size” OR “infarct size, brain” OR “brain infarction size” OR “brain stem infarct” OR “brain stem infarctions” OR “brainstem infarct” OR “brainstem infarction” OR “infarction, brain stem” OR “brain stem infarction” OR “cadasil syndrome” OR “cerebral autosomal dominant arteriopathy with subcortical infarct and leucoencephalopathy” OR “cerebral autosomal dominant arteriopathy with subcortical infarct and leukoencephalopathy” OR “cerebral autosomal dominant arteriopathy with subcortical infarcts and leucoencephalopathy” OR “cerebral autosomal dominant arteriopathy with subcortical infarcts and leukoencephalopathy” OR “cadasil” OR “cerebellar infarct” OR “cerebellar infarction” OR “cerebellum infarct” OR “cerebellum infarction” OR “laci (lacunar infarction)” OR “lacunar cerebral infarct” OR “lacunar cerebral infarction” OR “lacunar infarct” OR “lacunar infarction” OR “migraine infarct” OR “migraine infarction” OR “migrainous infarct” OR “migrainous infarction” OR “dementia, multi-infarct” OR “dementia, multiinfarct” OR “dementia, vascular” OR “lacunar dementia” OR “multi-infarct dementia” OR “multi-infarction dementia” OR “multiinfarction dementia” OR “vascular dementia” OR “multiinfarct dementia” OR “acute brain ischaemia” OR “acute brain ischemia” OR “brain arterial insufficiency” OR “brain circulation disorder” OR “brain ischaemia” OR “cerebral blood circulation disorder” OR “cerebral blood flow disorder” OR “cerebral circulation disorder” OR “cerebral circulatory disorder” OR “cerebral ischaemia” OR “cerebral ischemia” OR “cerebrovascular circulation disorder” OR “cerebrovascular ischaemia” OR “cerebrovascular ischemia” OR “ischaemia cerebri” OR “ischaemic brain disease” OR “ischaemic encephalopathy” OR “ischemia cerebri” OR “ischemic brain disease” OR “ischemic encephalopathy” OR “neural ischaemia” OR “neural ischemia” OR “brain ischemia” OR “anterior circulation ischemic event” OR “anterior circulation ischemia” OR “anterior circulation brain infarct” OR “anterior circulation brain infarction” OR “anterior circulation infarct” OR “anterior circulation ischemic infarct” OR “anterior circulation ischemic infarction” OR “anterior circulatory infarct” OR “anterior circulatory infarction” OR “anterior circulation infarction” OR “paci (partial anterior circulation infarct)” OR “partial anterior circulation brain infarct” OR “partial anterior circulation brain infarction” OR “partial anterior circulation infarction” OR “partial anterior circulatory infarct” OR “partial anterior circulatory infarction” OR “partial anterior circulation infarct” OR “taci (total anterior circulation infarct)” OR “total anterior circulation brain infarct” OR “total anterior circulation brain infarction” OR “total anterior circulation infarction” OR “total anterior circulatory infarct” OR “total anterior circulatory infarction” OR “total anterior circulation infarct” OR “anterior circulation ischaemic stroke” OR “anterior circulation ischemic stroke” OR “anterior circulation stroke syndrome” OR “anterior circulatory stroke” OR “anterior circulation stroke” OR “posterior circulation ischemic event” OR “posterior circulation ischemia” OR “posterior circulation brain infarct” OR “posterior circulation brain infarction” OR “posterior circulation infarct” OR “posterior circulation ischemic infarct” OR “posterior circulation ischemic infarction” OR “posterior circulatory infarct” OR “posterior circulatory infarction” OR “posterior circulation infarction” OR “posterior circulation ischaemic stroke” OR “posterior circulation ischemic stroke” OR “posterior circulation stroke syndrome” OR “posterior circulatory stroke” OR “posterior circulation stroke” OR “brain ischaemic attack” OR “brain ischemic attack” OR “brain transient ischaemic attack” OR “brain transient ischemic attack” OR “cerebral ischaemia, transient” OR “cerebral ischemia, transient” OR “circulatory epilepsy” OR “epilepsy circulatory” OR “ischaemic attack” OR “ischaemic attack, transient” OR “ischaemic cerebral attack” OR “ischemic attack” OR “ischemic attack, transient” OR “ischemic cerebral attack” OR “mini-stroke” OR “tia” OR “transient brain ischaemia” OR “transient brain ischemia” OR “transient cerebral ischaemia” OR “transient cerebral ischemia” OR “transient ischaemic attack” OR “transient ischaemic seizure” OR “transient ischemic seizure” OR “transient ischemic attack” OR “arteriosclerosis cerebri” OR “atherosclerosis cerebri” OR “atherosclerosis, brain” OR “atherosclerotic cerebrovascular disease” OR “brain arterio sclerosis” OR “brain arteriosclerosis” OR “brain atherosclerosis” OR “cerebral arteriosclerosis” OR “cerebral atherosclerotic disease” OR “cerebral presclerosis” OR “cerebrovascular arteriosclerosis” OR “cerebrovascular atherosclerosis” OR “cerebrovascular sclerosis” OR “cerebrum atherosclerosis” OR “intra-cranial atherosclerosis” OR “intracranial arteriosclerosis” OR “intracranial atherosclerosis” OR “cerebral atherosclerosis” OR “accident, cerebrovascular” OR “acute cerebrovascular lesion” OR “acute focal cerebral vasculopathy” OR “acute stroke” OR “apoplectic stroke” OR “apoplexia” OR “apoplexy” OR “blood flow disturbance, brain” OR “brain accident” OR “brain attack” OR “brain blood flow disturbance” OR “brain insult” OR “brain insultus” OR “brain vascular accident” OR “cerebral apoplexia” OR “cerebral insult” OR “cerebral stroke” OR “cerebral vascular accident” OR “cerebral vascular insufficiency” OR “cerebrovascular arrest” OR “cerebrovascular failure” OR “cerebrovascular injury” OR “cerebrovascular insufficiency” OR “cerebrovascular insult” OR “cerebrum vascular accident” OR “cryptogenic stroke” OR “cva” OR “insultus cerebralis” OR “ischaemic seizure” OR “ischemic seizure” OR “thrombotic stroke” OR “cerebrovascular accident” OR “brain stem stroke” OR “brainstem stroke” OR “embolic stroke” OR “cardioembolic stroke” OR “ischaemic stroke” OR “ischemic stroke” OR “lacs (lacunar syndrome)” OR “lacunar cerebral stroke” OR “lacunar stroke syndrome” OR “lacunar syndrome” OR “stroke, lacunar” OR “lacunar stroke” OR “brain blood vessel malformation” OR “brain vascular anomaly” OR “brain vascular malformation” OR “cerebral vascular anomaly” OR “cerebral vascular malformation” OR “cerebrovascular anomaly” OR “cerebrum vascular malformation” OR “vascular malformation, cerebrum” OR “arteriovenous aneurysm, brain” OR “arteriovenous cerebral aneurysm” OR “arteriovenous fistula, brain” OR “brain aneurysm, arteriovenous” OR “brain arteriovenous aneurysm” OR “brain arteriovenous fistula” OR “brain arteriovenous shunt” OR “cerebral aneurysm, arteriovenous” OR “cerebral arteriovenous fistula” OR “cerebral arteriovenous malformation” OR “cerebral arteriovenous malformations” OR “intracranial arteriovenous malformations” OR “brain arteriovenous malformation” OR “meningeal angiomatosis” OR “cerebral angiomatosis” OR “leptomeningeal angiomatosis” OR “angiomatosis cutanea cerebralis” OR “encephalo trigeminal angiomatosis” OR “encephalo-trigeminal angiomatosis” OR “encephalofacial angiomatosis” OR “encephalotrigeminal angiomatosis” OR “krabbe weber dimitri disease” OR “milles syndrome” OR “schirmer syndrome” OR “sturge weber disease” OR “sturge weber krabbe syndrome” OR “sturge-kalischer-weber syndrome” OR “sturge-weber syndrome” OR “sturge weber syndrome” OR “aneurysm of the vein of galen” OR “aneurysms of the vein of galen” OR “galen vein aneurysm” OR “great brain vein aneurysm” OR “malformation of the vein of galen” OR “malformations of the vein of galen” OR “vein of galen aneurysm” OR “vein of galen aneurysmal malformation” OR “vein of galen aneurysmal malformations” OR “vein of galen aneurysms” OR “vein of galen arteriovenous malformation” OR “vein of galen arteriovenous malformations” OR “vein of galen malformations” OR “vgam (vein of galen aneurysmal malformation)” OR “vgm (vein of galen malformation)” OR “vein of galen malformation” OR “hypophyseal apoplexy” OR “hypophysis haemorrhage” OR “hypophysis hemorrhage” OR “pituitary apoplexy” OR “pituitary gland apoplexy” OR “hypophysis apoplexy” OR “lipohyalinitic degeneration” OR “lipohyalinotic degeneration” OR “lipohyalinotic lesion” OR “lipohyalinotic occlusive disease” OR “lipohyalinosis” OR “melas” OR “mitochondrial encephalomyopathy with lactic acidosis and stroke-like episodes” OR “mitochondrial encephalomyopathy, lactic acidosis and stroke-like episodes” OR “mitochondrial encephalomyopathy, lactic acidosis with stroke-like episodes” OR “mitochondrial myopathy, encephalopathy, lactic acidosis and stroke-like episodes” OR “myoencephalopathy, lactic acidosis, stroke” OR “syndrome melas” OR “melas syndrome” OR “mitochondrial myopathy and sideroblastic anemia” OR “mitochondrial myopathy, lactic acidosis and sideroblastic anemia” OR “mlasa” OR “myopathy, lactic acidosis and sideroblastic anemia” OR “mlasa syndrome” OR “brain vascular obstruction” OR “cerebro-vascular occlusion” OR “cerebro-vascular occlusive disease” OR “cerebrovascular disease, occlusive” OR “cerebrovascular obliteration” OR “cerebrovascular obstruction” OR “cerebrovascular occlusion” OR “cerebrovascular occlusion disease” OR “cerebrovascular occlusive disease” OR “occlusive cerebro-vascular disease” OR “occlusive cerebrovascular disease” OR “brain phlebothrombosis” OR “brain thromboembolism” OR “brain thrombosis” OR “cerebral thromboses” OR “cerebro-vascular thrombosis” OR “cerebrovascular thrombosis” OR “intracranial thromboses” OR “intracranial thrombosis” OR “thromboembolism, brain” OR “thrombosis cerebri” OR “thrombosis, intracranial” OR “cerebral thrombosis” OR “cavernous sinus thrombosis syndrome” OR “cavernous thrombosis” OR “foix syndrome” OR “sinus cavernosus thrombosis” OR “thrombosis, cavernous sinus” OR “thrombosis, sinus cavernosus” OR “cavernous sinus thrombosis” OR “cavernous sinus thrombo-phlebitis” OR “cavernous thrombophlebitis” OR “thrombophlebitis of the cavernous sinus” OR “cavernous sinus thrombophlebitis” OR “thrombosis, lateral sinus” OR “transverse sinus thrombosis” OR “lateral sinus thrombosis” OR “longitudinal sinus thrombosis” OR “sinus sagittalis thrombosis” OR “superior longitudinal sinus thrombosis” OR “superior sagittal sinus thrombosis” OR “thrombosis, sagittal sinus” OR “sagittal sinus thrombosis” OR “syndrome, sneddon” OR “sneddon syndrome” OR “posterior encephalopathy” OR “posterior leucoencephalopathy syndrome” OR “posterior leukoencephalopathy” OR “posterior leukoencephalopathy syndrome” OR “posterior reversible encephalopathy” OR “posterior reversible leucoencephalopathy” OR “posterior reversible leucoencephalopathy syndrome” OR “posterior reversible leukoencephalopathy” OR “posterior reversible leukoencephalopathy syndrome” OR “pres (posterior reversible encephalopathy syndrome)” OR “reversible posterior leucoencephalopathy” OR “reversible posterior leucoencephalopathy syndrome” OR “reversible posterior leukoencephalopathy” OR “reversible posterior leukoencephalopathy syndrome” OR “rpls (reversible posterior leucoencephalopathy syndrome)” OR “rpls (reversible posterior leukoencephalopathy syndrome)” OR “posterior reversible encephalopathy syndrome” OR “subcortical ischaemic depression” OR “subcortical ischemic depression” OR “vascular depression” OR “arterial vertebrobasillaris insufficiency” OR “brachial basilar insufficiency” OR “vertebral basilar insufficiency” OR “vertebro basilar insufficiency” OR “vertebrobasilar disease” OR “vertebrobasilar ischaemia” OR “vertebrobasilar ischemia” OR “vertebrobasilar syndrome” OR “vertebrobasilar insufficiency” (Topic)	379129
#3	#1 AND #2	269

CINAHL

**Table 5:** Search strings used in CINAHL

Search strings used in CINAHL		
#1	(MH "Pulse Wave Velocity")	517
#2	TI ( (“cardiac-ankle vascular index” OR “cardio ankle vascular index” OR “cardio-vascular ankle index” OR “cardioankle vascular index” OR “cardiovascular ankle index” OR “cavi (cardio- ankle vascular index)” OR “cardio-ankle vascular index” OR “pwv (pulse wave velocity)” OR “aorta pulse wave velocity” OR “aortal pulse wave velocity” OR “aortic pwv” OR “pulse wave velocity of aorta” OR “pulse wave velocity of the aorta” OR “aortic pulse wave velocity” OR “arterial pulse velocity” OR “arterial pwv” OR “arterial wave velocity” OR “artery pulse velocity” OR “artery pulse wave velocity” OR “arterial pulse wave velocity” OR “ankle-brachial pulse wave velocity” OR “bapwv (brachial-ankle pulse wave velocity)” OR “brachial-ankle pulse velocity” OR “brachial-ankle wave velocity” OR “brachial-ankle pulse wave velocity” OR “carotid-femoral apwv” OR “carotid-femoral wave velocity” OR “cf-pwv (carotid-femoral pulse wave velocity)” OR “femoral carotid pulse velocity” OR “femoral-carotid pulse wave velocity” OR “carotid-femoral pulse wave velocity” OR “carotid-radial pulse wave velocity (pwv-cr)” OR “pwv-cr (carotid- radial pulse wave velocity)” OR “carotid-radial pulse wave velocity”) ) OR AB ( (“cardiac-ankle vascular index” OR “cardio ankle vascular index” OR “cardio-vascular ankle index” OR “cardioankle vascular index” OR “cardiovascular ankle index” OR “cavi (cardio-ankle vascular index)” OR “cardio-ankle vascular index” OR “pwv (pulse wave velocity)” OR “aorta pulse wave velocity” OR “aortal pulse wave velocity” OR “aortic pwv” OR “pulse wave velocity of aorta” OR “pulse wave velocity of the aorta” OR “aortic pulse wave velocity” OR “arterial pulse velocity” OR “arterial pwv” OR “arterial wave velocity” OR “artery pulse velocity” OR “artery pulse wave velocity” OR “arterial pulse wave velocity” OR “ankle-brachial pulse wave velocity” OR “bapwv (brachial-ankle pulse wave velocity)” OR “brachial-ankle pulse velocity” OR “brachial-ankle wave velocity” OR “brachial-ankle pulse wave velocity” OR “carotid-femoral apwv” OR “carotid-femoral wave velocity” OR “cf-pwv (carotid-femoral pulse wave velocity)” OR “femoral carotid pulse velocity” OR “femoral-carotid pulse wave velocity” OR “carotid-femoral pulse wave velocity” OR “carotid-radial pulse wave velocity (pwv-cr)” OR “pwv-cr (carotid-radial pulse wave velocity)” OR “carotid-radial pulse wave velocity”) )	2077
#3	S1 OR S2	2410
#4	(MH "Cerebral Small Vessel Diseases+")	2026
#5	TI ( (“brain angiopathy” OR “brain circulation failure” OR “brain vascular disease” OR “brain vasculopathy” OR “cerebral angiopathy” OR “cerebral small vessel disease” OR “cerebral small vessel diseases” OR “cerebral vascular disease” OR “cerebral vascular disorder” OR “cerebral vascular disturbance” OR “cerebral vascular lesion” OR “cerebral vasculopathy” OR “cerebro- vascular damage” OR “cerebro-vascular disease” OR “cerebro-vascular disorder” OR “cerebro-vascular disturbance” OR “cerebro-vascular lesion” OR “cerebro-vascular pathology” OR “cerebro- vascular syndrome” OR “cerebro-vasculopathy” OR “cerebroangiopathy” OR “cerebrovascular damage” OR “cerebrovascular disorder” OR “cerebrovascular disorders” OR “cerebrovascular disturbance” OR “cerebrovascular lesion” OR “cerebrovascular pathology” OR “cerebrovascular syndrome” OR “cerebrovasculopathy” OR “cerebrovascular disease” OR “basal ganglia cerebrovascular disease” OR “basal ganglia haemorrhage” OR “basal ganglia hemorrhage” OR “basal ganglion cerebrovascular disease” OR “basal ganglion haemorrhage” OR “cerebrovascular disease, basal ganglion” OR “haemorrhage, basal ganglion” OR “hemorrhage, basal ganglion” OR “basal ganglion hemorrhage” OR “haemorrhage, putamen” OR “hemorrhage, putamen” OR “putamen haematoma” OR “putamen haemorrhage” OR “putamen hematoma” OR “putamen hemorrhage” OR “putaminal haematoma” OR “putaminal haemorrhage” OR “putaminal hematoma” OR “putaminal hemorrhage” OR “brain haematoma” OR “cerebral haematoma” OR “cerebral hematoma” OR “haematoma, brain” OR “hematoma, brain” OR “interhemispheric haematoma” OR “interhemispheric hematoma” OR “intracerebral haematoma” OR “intracerebral hematoma” OR “intracranial haematoma” OR “intracranial hematoma” OR “brain hematoma” OR “acute subdural haematoma” OR “acute subdural hematoma” OR “chronic subdural haematoma” OR “chronic subdural haematomata” OR “chronic subdural hematoma” OR “chronic subdural hematomata” OR “haematoma, subdural” OR “haematoma, subdural, acute” OR “haematoma, subdural, chronic” OR “haematoma, subdural, intracranial” OR “haemorrhage, subdural” OR “haemorrhagic pachymeningitis” OR “hematoma, subdural” OR “hematoma, subdural, acute” OR “hematoma, subdural, chronic” OR “hematoma, subdural, intracranial” OR “hemorrhage, subdural” OR “hemorrhagic pachymeningitis” OR “intracranial subdural haematoma” OR “intracranial subdural haematomas” OR “intracranial subdural haematomata” OR “intracranial subdural hematoma” OR “intracranial subdural hematomas” OR “intracranial subdural hematomata” OR “pachymeningiosis haemorrhagica interna” OR “pachymeningitis haemorrhagica” OR “subdural bleeding” OR “subdural haematoma” OR “subdural haemorrhage” OR “subdural hemorrhage” OR “subepidural haematoma” OR “subepidural hematoma” OR “subdural hematoma” OR “bleeding, corpus callosum” OR “brain bleeding” OR “brain haemorrhage” OR “brain haemorrhage, traumatic” OR “brain hemorrhage, traumatic” OR “brain microhaemorrhage” OR “brain microhemorrhage” OR “brain stem haemorrhage, traumatic” OR “brain stem hemorrhage, traumatic” OR “cerebral haemorrhage” OR “cerebral haemorrhage, traumatic” OR “cerebral hemorrhage” OR “cerebral hemorrhage, traumatic” OR “cerebral microbleed” OR “corpus callosum bleeding” OR “corpus callosum haemorrhage” OR “corpus callosum hemorrhage” OR “encephalorrhagia” OR “haemorrhage, brain” OR “haemorrhage, intracranial” OR “haemorrhagic apoplexy” OR “haemorrhagic stroke” OR “haemorrhagic stroke intracerebral bleeding” OR “hematencephalon” OR “hemorrhage, brain” OR “hemorrhage, intracranial” OR “hemorrhagic apoplexy” OR “hemorrhagic stroke” OR “hemorrhagic stroke intracerebral bleeding” OR “hypertensive intracranial haemorrhage” OR “hypertensive intracranial hemorrhage” OR “intracerebral bleeding” OR “intracerebral haemorrhage” OR “intracerebral hemorrhage” OR “intracortical haemorrhage” OR “intracortical hemorrhage” OR “intracranial bleeding” OR “intracranial haemorrhage” OR “intracranial haemorrhage, hypertensive” OR “intracranial haemorrhage, traumatic” OR “intracranial haemorrhages” OR “intracranial hemorrhage” OR “intracranial hemorrhage, hypertensive” OR “intracranial hemorrhage, traumatic” OR “intracranial hemorrhages” OR “intraventricular haemorrhage” OR “intraventricular hemorrhage” OR “periventricular haemorrhage” OR “periventricular hemorrhage” OR “posterior fossa haemorrhage” OR “posterior fossa hemorrhage” OR “traumatic brain haemorrhage” OR “traumatic brain hemorrhage” OR “traumatic brain stem haemorrhage” OR “traumatic brain stem hemorrhage” OR “traumatic cerebral haemorrhage” OR “traumatic cerebral hemorrhage” OR “traumatic intracranial haemorrhage” OR “traumatic intracranial hemorrhage” OR “brain hemorrhage” OR “brain ventricle bleeding” OR “brain ventricle haemorrhage” OR “cerebral intraventricular hemorrhage” OR “brain ventricle hemorrhage” OR “cerebellar haemorrhage” OR “cerebellar hemorrhage” OR “cerebellum haemorrhage” OR “haemorrhage, cerebellum” OR “hemorrhage, cerebellum” OR “cerebellum hemorrhage” OR “massive cerebral bleeding” OR “massive cerebral haemorrhage” OR “massive cerebral hemorrhage” OR “massive intracerebral bleeding” OR “massive intracerebral haemorrhage” OR “massive intracerebral hemorrhage” OR “aneurysmal subarachnoid haemorrhage” OR “aneurysmal subarachnoid hemorrhage” OR “arachnoid haemorrhage, brain” OR “arachnoid hemorrhage, brain” OR “arachnoidal bleeding” OR “arachnoidal haemorrhage” OR “arachnoidal haemorrhage, brain” OR “arachnoidal hemorrhage” OR “arachnoidal hemorrhage, brain” OR “bleeding, subarachnoid” OR “brain arachnoid haemorrhage” OR “brain arachnoid hemorrhage” OR “haemorrhage, subarachnoid” OR “hemorrhage, subarachnoid” OR “spontaneous subarachnoid haemorrhage” OR “spontaneous subarachnoid hemorrhage” OR “subarachnoid bleeding” OR “subarachnoid blood” OR “subarachnoid haematoma” OR “subarachnoid haemorrhage” OR “subarachnoid haemorrhage, brain” OR “subarachnoid haemorrhage, traumatic” OR “subarachnoid hematoma” OR “subarachnoid hemorrhage, brain” OR “subarachnoid hemorrhage, traumatic” OR “subarachnoid hemorrhagia” OR “subarachnoidal bleeding” OR “subarachnoidal haemorrhage” OR “subarachnoidal hemorrhage” OR “traumatic subarachnoid haemorrhage” OR “traumatic subarachnoid hemorrhage” OR “subarachnoid hemorrhage” OR “brain cortex infarct” OR “brain cortex infarction” OR “brain infarct” OR “cerebral cortex infarct” OR “cerebral cortex infarction” OR “cerebral infarct” OR “cerebral infarction” OR “cerebrovascular infarct” OR “cerebrovascular infarction” OR “cortical infarct” OR “cortical infarction” OR “hemisphere infarct” OR “hemisphere infarction” OR “hemispheric infarct” OR “hemispheric infarction” OR “infarction, brain” OR “silent brain infarction” OR “brain infarction” OR “brain infarct size” OR “cerebral infarct size” OR “cerebral infarction size” OR “cortical infarct size” OR “cortical infarction size” OR “infarct size, brain” OR “brain infarction size” OR “brain stem infarct” OR “brain stem infarctions” OR “brainstem infarct” OR “brainstem infarction” OR “infarction, brain stem” OR “brain stem infarction” OR “cadasil syndrome” OR “cerebral autosomal dominant arteriopathy with subcortical infarct and leucoencephalopathy” OR “cerebral autosomal dominant arteriopathy with subcortical infarct and leukoencephalopathy” OR “cerebral autosomal dominant arteriopathy with subcortical infarcts and leucoencephalopathy” OR “cerebral autosomal dominant arteriopathy with subcortical infarcts and leukoencephalopathy” OR “cadasil” OR “cerebellar infarct” OR “cerebellar infarction” OR “cerebellum infarct” OR “cerebellum infarction” OR “laci (lacunar infarction)” OR “lacunar cerebral infarct” OR “lacunar cerebral infarction” OR “lacunar infarct” OR “lacunar infarction” OR “migraine infarct” OR “migraine infarction” OR “migrainous infarct” OR “migrainous infarction” OR “dementia, multi-infarct” OR “dementia, multiinfarct” OR “dementia, vascular” OR “lacunar dementia” OR “multi- infarct dementia” OR “multi-infarction dementia” OR “multiinfarction dementia” OR “vascular dementia” OR “multiinfarct dementia” OR “acute brain ischaemia” OR “acute brain ischemia” OR “brain arterial insufficiency” OR “brain circulation disorder” OR “brain ischaemia” OR “cerebral blood circulation disorder” OR “cerebral blood flow disorder” OR “cerebral circulation disorder” OR “cerebral circulatory disorder” OR “cerebral ischaemia” OR “cerebral ischemia” OR “cerebrovascular circulation disorder” OR “cerebrovascular ischaemia” OR “cerebrovascular ischemia” OR “ischaemia cerebri” OR “ischaemic brain disease” OR “ischaemic encephalopathy” OR “ischemia cerebri” OR “ischemic brain disease” OR “ischemic encephalopathy” OR “neural ischaemia” OR “neural ischemia” OR “brain ischemia” OR “anterior circulation ischemic event” OR “anterior circulation ischemia” OR “anterior circulation brain infarct” OR “anterior circulation brain infarction” OR “anterior circulation infarct” OR “anterior circulation ischemic infarct” OR “anterior circulation ischemic infarction” OR “anterior circulatory infarct” OR “anterior circulatory infarction” OR “anterior circulation infarction” OR “paci (partial anterior circulation infarct)” OR “partial anterior circulation brain infarct” OR “partial anterior circulation brain infarction” OR “partial anterior circulation infarction” OR “partial anterior circulatory infarct” OR “partial anterior circulatory infarction” OR “partial anterior circulation infarct” OR “taci (total anterior circulation infarct)” OR “total anterior circulation brain infarct” OR “total anterior circulation brain infarction” OR “total anterior circulation infarction” OR “total anterior circulatory infarct” OR “total anterior circulatory infarction” OR “total anterior circulation infarct” OR “anterior circulation ischaemic stroke” OR “anterior circulation ischemic stroke” OR “anterior circulation stroke syndrome” OR “anterior circulatory stroke” OR “anterior circulation stroke” OR “posterior circulation ischemic event” OR “posterior circulation ischemia” OR “posterior circulation brain infarct” OR “posterior circulation brain infarction” OR “posterior circulation infarct” OR “posterior circulation ischemic infarct” OR “posterior circulation ischemic infarction” OR “posterior circulatory infarct” OR “posterior circulatory infarction” OR “posterior circulation infarction” OR “posterior circulation ischaemic stroke” OR “posterior circulation ischemic stroke” OR “posterior circulation stroke syndrome” OR “posterior circulatory stroke” OR “posterior circulation stroke” OR “brain ischaemic attack” OR “brain ischemic attack” OR “brain transient ischaemic attack” OR “brain transient ischemic attack” OR “cerebral ischaemia, transient” OR “cerebral ischemia, transient” OR “circulatory epilepsy” OR “epilepsy circulatory” OR “ischaemic attack” OR “ischaemic attack, transient” OR “ischaemic cerebral attack” OR “ischemic attack” OR “ischemic attack, transient” OR “ischemic cerebral attack” OR “mini-stroke” OR “tia” OR “transient brain ischaemia” OR “transient brain ischemia” OR “transient cerebral ischaemia” OR “transient cerebral ischemia” OR “transient ischaemic attack” OR “transient ischaemic seizure” OR “transient ischemic seizure” OR “transient ischemic attack” OR “arteriosclerosis cerebri” OR “atherosclerosis cerebri” OR “atherosclerosis, brain” OR “atherosclerotic cerebrovascular disease” OR “brain arterio sclerosis” OR “brain arteriosclerosis” OR “brain atherosclerosis” OR “cerebral arteriosclerosis” OR “cerebral atherosclerotic disease” OR “cerebral presclerosis” OR “cerebrovascular arteriosclerosis” OR “cerebrovascular atherosclerosis” OR “cerebrovascular sclerosis” OR “cerebrum atherosclerosis” OR “intra-cranial atherosclerosis” OR “intracranial arteriosclerosis” OR “intracranial atherosclerosis” OR “cerebral atherosclerosis” OR “accident, cerebrovascular” OR “acute cerebrovascular lesion” OR “acute focal cerebral vasculopathy” OR “acute stroke” OR “apoplectic stroke” OR “apoplexia” OR “apoplexy” OR “blood flow disturbance, brain” OR “brain accident” OR “brain attack” OR “brain blood flow disturbance” OR “brain insult” OR “brain insultus” OR “brain vascular accident” OR “cerebral apoplexia” OR “cerebral insult” OR “cerebral stroke” OR “cerebral vascular accident” OR “cerebral vascular insufficiency” OR “cerebrovascular arrest” OR “cerebrovascular failure” OR “cerebrovascular injury” OR “cerebrovascular insufficiency” OR “cerebrovascular insult” OR “cerebrum vascular accident” OR “cryptogenic stroke” OR “cva” OR “insultus cerebralis” OR “ischaemic seizure” OR “ischemic seizure” OR “thrombotic stroke” OR “cerebrovascular accident” OR “brain stem stroke” OR “brainstem stroke” OR “embolic stroke” OR “cardioembolic stroke” OR “ischaemic stroke” OR “ischemic stroke” OR “lacs (lacunar syndrome)” OR “lacunar cerebral stroke” OR “lacunar stroke syndrome” OR “lacunar syndrome” OR “stroke, lacunar” OR “lacunar stroke” OR “brain blood vessel malformation” OR “brain vascular anomaly” OR “brain vascular malformation” OR “cerebral vascular anomaly” OR “cerebral vascular malformation” OR “cerebrovascular anomaly” OR “cerebrum vascular malformation” OR “vascular malformation, cerebrum” OR “arteriovenous aneurysm, brain” OR “arteriovenous cerebral aneurysm” OR “arteriovenous fistula, brain” OR “brain aneurysm, arteriovenous” OR “brain arteriovenous aneurysm” OR “brain arteriovenous fistula” OR “brain arteriovenous shunt” OR “cerebral aneurysm, arteriovenous” OR “cerebral arteriovenous fistula” OR “cerebral arteriovenous malformation” OR “cerebral arteriovenous malformations” OR “intracranial arteriovenous malformations” OR “brain arteriovenous malformation” OR “meningeal angiomatosis” OR “cerebral angiomatosis” OR “leptomeningeal angiomatosis” OR “angiomatosis cutanea cerebralis” OR “encephalo trigeminal angiomatosis” OR “encephalo-trigeminal angiomatosis” OR “encephalofacial angiomatosis” OR “encephalotrigeminal angiomatosis” OR “krabbe weber dimitri disease” OR “milles syndrome” OR “schirmer syndrome” OR “sturge weber disease” OR “sturge weber krabbe syndrome” OR “sturge-kalischer-weber syndrome” OR “sturge-weber syndrome” OR “sturge weber syndrome” OR “aneurysm of the vein of galen” OR “aneurysms of the vein of galen” OR “galen vein aneurysm” OR “great brain vein aneurysm” OR “malformation of the vein of galen” OR “malformations of the vein of galen” OR “vein of galen aneurysm” OR “vein of galen aneurysmal malformation” OR “vein of galen aneurysmal malformations” OR “vein of galen aneurysms” OR “vein of galen arteriovenous malformation” OR “vein of galen arteriovenous malformations” OR “vein of galen malformations” OR “vgam (vein of galen aneurysmal malformation)” OR “vgm (vein of galen malformation)” OR “vein of galen malformation” OR “hypophyseal apoplexy” OR “hypophysis haemorrhage” OR “hypophysis hemorrhage” OR “pituitary apoplexy” OR “pituitary gland apoplexy” OR “hypophysis apoplexy” OR “lipohyalinitic degeneration” OR “lipohyalinotic degeneration” OR “lipohyalinotic lesion” OR “lipohyalinotic occlusive disease” OR “lipohyalinosis” OR “melas” OR “mitochondrial encephalomyopathy with lactic acidosis and stroke-like episodes” OR “mitochondrial encephalomyopathy, lactic acidosis and stroke- like episodes” OR “mitochondrial encephalomyopathy, lactic acidosis with stroke-like episodes” OR “mitochondrial myopathy, encephalopathy, lactic acidosis and stroke-like episodes” OR “myoencephalopathy, lactic acidosis, stroke” OR “syndrome melas” OR “melas syndrome” OR “mitochondrial myopathy and sideroblastic anemia” OR “mitochondrial myopathy, lactic acidosis and sideroblastic anemia” OR “mlasa” OR “myopathy, lactic acidosis and sideroblastic anemia” OR “mlasa syndrome” OR “brain vascular obstruction” OR “cerebro-vascular occlusion” OR “cerebro-vascular occlusive disease” OR “cerebrovascular disease, occlusive” OR “cerebrovascular obliteration” OR “cerebrovascular obstruction” OR “cerebrovascular occlusion” OR “cerebrovascular occlusion disease” OR “cerebrovascular occlusive disease” OR “occlusive cerebro-vascular disease” OR “occlusive cerebrovascular disease” OR “brain phlebothrombosis” OR “brain thromboembolism” OR “brain thrombosis” OR “cerebral thromboses” OR “cerebro-vascular thrombosis” OR “cerebrovascular thrombosis” OR “intracranial thromboses” OR “intracranial thrombosis” OR “thromboembolism, brain” OR “thrombosis cerebri” OR “thrombosis, intracranial” OR “cerebral thrombosis” OR “cavernous sinus thrombosis syndrome” OR “cavernous thrombosis” OR “foix syndrome” OR “sinus cavernosus thrombosis” OR “thrombosis, cavernous sinus” OR “thrombosis, sinus cavernosus” OR “cavernous sinus thrombosis” OR “cavernous sinus thrombo-phlebitis” OR “cavernous thrombophlebitis” OR “thrombophlebitis of the cavernous sinus” OR “cavernous sinus thrombophlebitis” OR “thrombosis, lateral sinus” OR “transverse sinus thrombosis” OR “lateral sinus thrombosis” OR “longitudinal sinus thrombosis” OR “sinus sagittalis thrombosis” OR “superior longitudinal sinus thrombosis” OR “superior sagittal sinus thrombosis” OR “thrombosis, sagittal sinus” OR “sagittal sinus thrombosis” OR “syndrome, sneddon” OR “sneddon syndrome” OR “posterior encephalopathy” OR “posterior leucoencephalopathy syndrome” OR “posterior leukoencephalopathy” OR “posterior leukoencephalopathy syndrome” OR “posterior reversible encephalopathy” OR “posterior reversible leucoencephalopathy” OR “posterior reversible leucoencephalopathy syndrome” OR “posterior reversible leukoencephalopathy” OR “posterior reversible leukoencephalopathy syndrome” OR “pres (posterior reversible encephalopathy syndrome)” OR “reversible posterior leucoencephalopathy” OR “reversible posterior leucoencephalopathy syndrome” OR “reversible posterior leukoencephalopathy” OR “reversible posterior leukoencephalopathy syndrome” OR “rpls (reversible posterior leucoencephalopathy syndrome)” OR “rpls (reversible posterior leukoencephalopathy syndrome)” OR “posterior reversible encephalopathy syndrome” OR “subcortical ischaemic depression” OR “subcortical ischemic depression” OR “vascular depression” OR “arterial vertebrobasillaris insufficiency” OR “brachial basilar insufficiency” OR “vertebral basilar insufficiency” OR “vertebro basilar insufficiency” OR “vertebrobasilar disease” OR “vertebrobasilar ischaemia” OR “vertebrobasilar ischemia” OR “vertebrobasilar syndrome” OR “vertebrobasilar insufficiency”) ) OR AB ( (“brain angiopathy” OR “brain circulation failure” OR “brain vascular disease” OR “brain vasculopathy” OR “cerebral angiopathy” OR “cerebral small vessel disease” OR “cerebral small vessel diseases” OR “cerebral vascular disease” OR “cerebral vascular disorder” OR “cerebral vascular disturbance” OR “cerebral vascular lesion” OR “cerebral vasculopathy” OR “cerebro-vascular damage” OR “cerebro-vascular disease” OR “cerebro- vascular disorder” OR “cerebro-vascular disturbance” OR “cerebro-vascular lesion” OR “cerebro-vascular pathology” OR “cerebro-vascular syndrome” OR “cerebro-vasculopathy” OR “cerebroangiopathy” OR “cerebrovascular damage” OR “cerebrovascular disorder” OR “cerebrovascular disorders” OR “cerebrovascular disturbance” OR “cerebrovascular lesion” OR “cerebrovascular pathology” OR “cerebrovascular syndrome” OR “cerebrovasculopathy” OR “cerebrovascular disease” OR “basal ganglia cerebrovascular disease” OR “basal ganglia haemorrhage” OR “basal ganglia hemorrhage” OR “basal ganglion cerebrovascular disease” OR “basal ganglion haemorrhage” OR “cerebrovascular disease, basal ganglion” OR “haemorrhage, basal ganglion” OR “hemorrhage, basal ganglion” OR “basal ganglion hemorrhage” OR “haemorrhage, putamen” OR “hemorrhage, putamen” OR “putamen haematoma” OR “putamen haemorrhage” OR “putamen hematoma” OR “putamen hemorrhage” OR “putaminal haematoma” OR “putaminal haemorrhage” OR “putaminal hematoma” OR “putaminal hemorrhage” OR “brain haematoma” OR “cerebral haematoma” OR “cerebral hematoma” OR “haematoma, brain” OR “hematoma, brain” OR “interhemispheric haematoma” OR “interhemispheric hematoma” OR “intracerebral haematoma” OR “intracerebral hematoma” OR “intracranial haematoma” OR “intracranial hematoma” OR “brain hematoma” OR “acute subdural haematoma” OR “acute subdural hematoma” OR “chronic subdural haematoma” OR “chronic subdural haematomata” OR “chronic subdural hematoma” OR “chronic subdural hematomata” OR “haematoma, subdural” OR “haematoma, subdural, acute” OR “haematoma, subdural, chronic” OR “haematoma, subdural, intracranial” OR “haemorrhage, subdural” OR “haemorrhagic pachymeningitis” OR “hematoma, subdural” OR “hematoma, subdural, acute” OR “hematoma, subdural, chronic” OR “hematoma, subdural, intracranial” OR “hemorrhage, subdural” OR “hemorrhagic pachymeningitis” OR “intracranial subdural haematoma” OR “intracranial subdural haematomas” OR “intracranial subdural haematomata” OR “intracranial subdural hematoma” OR “intracranial subdural hematomas” OR “intracranial subdural hematomata” OR “pachymeningiosis haemorrhagica interna” OR “pachymeningitis haemorrhagica” OR “subdural bleeding” OR “subdural haematoma” OR “subdural haemorrhage” OR “subdural hemorrhage” OR “subepidural haematoma” OR “subepidural hematoma” OR “subdural hematoma” OR “bleeding, corpus callosum” OR “brain bleeding” OR “brain haemorrhage” OR “brain haemorrhage, traumatic” OR “brain hemorrhage, traumatic” OR “brain microhaemorrhage” OR “brain microhemorrhage” OR “brain stem haemorrhage, traumatic” OR “brain stem hemorrhage, traumatic” OR “cerebral haemorrhage” OR “cerebral haemorrhage, traumatic” OR “cerebral hemorrhage” OR “cerebral hemorrhage, traumatic” OR “cerebral microbleed” OR “corpus callosum bleeding” OR “corpus callosum haemorrhage” OR “corpus callosum hemorrhage” OR “encephalorrhagia” OR “haemorrhage, brain” OR “haemorrhage, intracranial” OR “haemorrhagic apoplexy” OR “haemorrhagic stroke” OR “haemorrhagic stroke intracerebral bleeding” OR “hematencephalon” OR “hemorrhage, brain” OR “hemorrhage, intracranial” OR “hemorrhagic apoplexy” OR “hemorrhagic stroke” OR “hemorrhagic stroke intracerebral bleeding” OR “hypertensive intracranial haemorrhage” OR “hypertensive intracranial hemorrhage” OR “intracerebral bleeding” OR “intracerebral haemorrhage” OR “intracerebral hemorrhage” OR “intracortical haemorrhage” OR “intracortical hemorrhage” OR “intracranial bleeding” OR “intracranial haemorrhage” OR “intracranial haemorrhage, hypertensive” OR “intracranial haemorrhage, traumatic” OR “intracranial haemorrhages” OR “intracranial hemorrhage” OR “intracranial hemorrhage, hypertensive” OR “intracranial hemorrhage, traumatic” OR “intracranial hemorrhages” OR “intraventricular haemorrhage” OR “intraventricular hemorrhage” OR “periventricular haemorrhage” OR “periventricular hemorrhage” OR “posterior fossa haemorrhage” OR “posterior fossa hemorrhage” OR “traumatic brain haemorrhage” OR “traumatic brain hemorrhage” OR “traumatic brain stem haemorrhage” OR “traumatic brain stem hemorrhage” OR “traumatic cerebral haemorrhage” OR “traumatic cerebral hemorrhage” OR “traumatic intracranial haemorrhage” OR “traumatic intracranial hemorrhage” OR “brain hemorrhage” OR “brain ventricle bleeding” OR “brain ventricle haemorrhage” OR “cerebral intraventricular hemorrhage” OR “brain ventricle hemorrhage” OR “cerebellar haemorrhage” OR “cerebellar hemorrhage” OR “cerebellum haemorrhage” OR “haemorrhage, cerebellum” OR “hemorrhage, cerebellum” OR “cerebellum hemorrhage” OR “massive cerebral bleeding” OR “massive cerebral haemorrhage” OR “massive cerebral hemorrhage” OR “massive intracerebral bleeding” OR “massive intracerebral haemorrhage” OR “massive intracerebral hemorrhage” OR “aneurysmal subarachnoid haemorrhage” OR “aneurysmal subarachnoid hemorrhage” OR “arachnoid haemorrhage, brain” OR “arachnoid hemorrhage, brain” OR “arachnoidal bleeding” OR “arachnoidal haemorrhage” OR “arachnoidal haemorrhage, brain” OR “arachnoidal hemorrhage” OR “arachnoidal hemorrhage, brain” OR “bleeding, subarachnoid” OR “brain arachnoid haemorrhage” OR “brain arachnoid hemorrhage” OR “haemorrhage, subarachnoid” OR “hemorrhage, subarachnoid” OR “spontaneous subarachnoid haemorrhage” OR “spontaneous subarachnoid hemorrhage” OR “subarachnoid bleeding” OR “subarachnoid blood” OR “subarachnoid haematoma” OR “subarachnoid haemorrhage” OR “subarachnoid haemorrhage, brain” OR “subarachnoid haemorrhage, traumatic” OR “subarachnoid hematoma” OR “subarachnoid hemorrhage, brain” OR “subarachnoid hemorrhage, traumatic” OR “subarachnoid hemorrhagia” OR “subarachnoidal bleeding” OR “subarachnoidal haemorrhage” OR “subarachnoidal hemorrhage” OR “traumatic subarachnoid haemorrhage” OR “traumatic subarachnoid hemorrhage” OR “subarachnoid hemorrhage” OR “brain cortex infarct” OR “brain cortex infarction” OR “brain infarct” OR “cerebral cortex infarct” OR “cerebral cortex infarction” OR “cerebral infarct” OR “cerebral infarction” OR “cerebrovascular infarct” OR “cerebrovascular infarction” OR “cortical infarct” OR “cortical infarction” OR “hemisphere infarct” OR “hemisphere infarction” OR “hemispheric infarct” OR “hemispheric infarction” OR “infarction, brain” OR “silent brain infarction” OR “brain infarction” OR “brain infarct size” OR “cerebral infarct size” OR “cerebral infarction size” OR “cortical infarct size” OR “cortical infarction size” OR “infarct size, brain” OR “brain infarction size” OR “brain stem infarct” OR “brain stem infarctions” OR “brainstem infarct” OR “brainstem infarction” OR “infarction, brain stem” OR “brain stem infarction” OR “cadasil syndrome” OR “cerebral autosomal dominant arteriopathy with subcortical infarct and leucoencephalopathy” OR “cerebral autosomal dominant arteriopathy with subcortical infarct and leukoencephalopathy” OR “cerebral autosomal dominant arteriopathy with subcortical infarcts and leucoencephalopathy” OR “cerebral autosomal dominant arteriopathy with subcortical infarcts and leukoencephalopathy” OR “cadasil” OR “cerebellar infarct” OR “cerebellar infarction” OR “cerebellum infarct” OR “cerebellum infarction” OR “laci (lacunar infarction)” OR “lacunar cerebral infarct” OR “lacunar cerebral infarction” OR “lacunar infarct” OR “lacunar infarction” OR “migraine infarct” OR “migraine infarction” OR “migrainous infarct” OR “migrainous infarction” OR “dementia, multi-infarct” OR “dementia, multiinfarct” OR “dementia, vascular” OR “lacunar dementia” OR “multi-infarct dementia” OR “multi-infarction dementia” OR “multiinfarction dementia” OR “vascular dementia” OR “multiinfarct dementia” OR “acute brain ischaemia” OR “acute brain ischemia” OR “brain arterial insufficiency” OR “brain circulation disorder” OR “brain ischaemia” OR “cerebral blood circulation disorder” OR “cerebral blood flow disorder” OR “cerebral circulation disorder” OR “cerebral circulatory disorder” OR “cerebral ischaemia” OR “cerebral ischemia” OR “cerebrovascular circulation disorder” OR “cerebrovascular ischaemia” OR “cerebrovascular ischemia” OR “ischaemia cerebri” OR “ischaemic brain disease” OR “ischaemic encephalopathy” OR “ischemia cerebri” OR “ischemic brain disease” OR “ischemic encephalopathy” OR “neural ischaemia” OR “neural ischemia” OR “brain ischemia” OR “anterior circulation ischemic event” OR “anterior circulation ischemia” OR “anterior circulation brain infarct” OR “anterior circulation brain infarction” OR “anterior circulation infarct” OR “anterior circulation ischemic infarct” OR “anterior circulation ischemic infarction” OR “anterior circulatory infarct” OR “anterior circulatory infarction” OR “anterior circulation infarction” OR “paci (partial anterior circulation infarct)” OR “partial anterior circulation brain infarct” OR “partial anterior circulation brain infarction” OR “partial anterior circulation infarction” OR “partial anterior circulatory infarct” OR “partial anterior circulatory infarction” OR “partial anterior circulation infarct” OR “taci (total anterior circulation infarct)” OR “total anterior circulation brain infarct” OR “total anterior circulation brain infarction” OR “total anterior circulation infarction” OR “total anterior circulatory infarct” OR “total anterior circulatory infarction” OR “total anterior circulation infarct” OR “anterior circulation ischaemic stroke” OR “anterior circulation ischemic stroke” OR “anterior circulation stroke syndrome” OR “anterior circulatory stroke” OR “anterior circulation stroke” OR “posterior circulation ischemic event” OR “posterior circulation ischemia” OR “posterior circulation brain infarct” OR “posterior circulation brain infarction” OR “posterior circulation infarct” OR “posterior circulation ischemic infarct” OR “posterior circulation ischemic infarction” OR “posterior circulatory infarct” OR “posterior circulatory infarction” OR “posterior circulation infarction” OR “posterior circulation ischaemic stroke” OR “posterior circulation ischemic stroke” OR “posterior circulation stroke syndrome” OR “posterior circulatory stroke” OR “posterior circulation stroke” OR “brain ischaemic attack” OR “brain ischemic attack” OR “brain transient ischaemic attack” OR “brain transient ischemic attack” OR “cerebral ischaemia, transient” OR “cerebral ischemia, transient” OR “circulatory epilepsy” OR “epilepsy circulatory” OR “ischaemic attack” OR “ischaemic attack, transient” OR “ischaemic cerebral attack” OR “ischemic attack” OR “ischemic attack, transient” OR “ischemic cerebral attack” OR “mini-stroke” OR “tia” OR “transient brain ischaemia” OR “transient brain ischemia” OR “transient cerebral ischaemia” OR “transient cerebral ischemia” OR “transient ischaemic attack” OR “transient ischaemic seizure” OR “transient ischemic seizure” OR “transient ischemic attack” OR “arteriosclerosis cerebri” OR “atherosclerosis cerebri” OR “atherosclerosis, brain” OR “atherosclerotic cerebrovascular disease” OR “brain arterio sclerosis” OR “brain arteriosclerosis” OR “brain atherosclerosis” OR “cerebral arteriosclerosis” OR “cerebral atherosclerotic disease” OR “cerebral presclerosis” OR “cerebrovascular arteriosclerosis” OR “cerebrovascular atherosclerosis” OR “cerebrovascular sclerosis” OR “cerebrum atherosclerosis” OR “intra-cranial atherosclerosis” OR “intracranial arteriosclerosis” OR “intracranial atherosclerosis” OR “cerebral atherosclerosis” OR “accident, cerebrovascular” OR “acute cerebrovascular lesion” OR “acute focal cerebral vasculopathy” OR “acute stroke” OR “apoplectic stroke” OR “apoplexia” OR “apoplexy” OR “blood flow disturbance, brain” OR “brain accident” OR “brain attack” OR “brain blood flow disturbance” OR “brain insult” OR “brain insultus” OR “brain vascular accident” OR “cerebral apoplexia” OR “cerebral insult” OR “cerebral stroke” OR “cerebral vascular accident” OR “cerebral vascular insufficiency” OR “cerebrovascular arrest” OR “cerebrovascular failure” OR “cerebrovascular injury” OR “cerebrovascular insufficiency” OR “cerebrovascular insult” OR “cerebrum vascular accident” OR “cryptogenic stroke” OR “cva” OR “insultus cerebralis” OR “ischaemic seizure” OR “ischemic seizure” OR “thrombotic stroke” OR “cerebrovascular accident” OR “brain stem stroke” OR “brainstem stroke” OR “embolic stroke” OR “cardioembolic stroke” OR “ischaemic stroke” OR “ischemic stroke” OR “lacs (lacunar syndrome)” OR “lacunar cerebral stroke” OR “lacunar stroke syndrome” OR “lacunar syndrome” OR “stroke, lacunar” OR “lacunar stroke” OR “brain blood vessel malformation” OR “brain vascular anomaly” OR “brain vascular malformation” OR “cerebral vascular anomaly” OR “cerebral vascular malformation” OR “cerebrovascular anomaly” OR “cerebrum vascular malformation” OR “vascular malformation, cerebrum” OR “arteriovenous aneurysm, brain” OR “arteriovenous cerebral aneurysm” OR “arteriovenous fistula, brain” OR “brain aneurysm, arteriovenous” OR “brain arteriovenous aneurysm” OR “brain arteriovenous fistula” OR “brain arteriovenous shunt” OR “cerebral aneurysm, arteriovenous” OR “cerebral arteriovenous fistula” OR “cerebral arteriovenous malformation” OR “cerebral arteriovenous malformations” OR “intracranial arteriovenous malformations” OR “brain arteriovenous malformation” OR “meningeal angiomatosis” OR “cerebral angiomatosis” OR “leptomeningeal angiomatosis” OR “angiomatosis cutanea cerebralis” OR “encephalo trigeminal angiomatosis” OR “encephalo-trigeminal angiomatosis” OR “encephalofacial angiomatosis” OR “encephalotrigeminal angiomatosis” OR “krabbe weber dimitri disease” OR “milles syndrome” OR “schirmer syndrome” OR “sturge weber disease” OR “sturge weber krabbe syndrome” OR “sturge-kalischer-weber syndrome” OR “sturge-weber syndrome” OR “sturge weber syndrome” OR “aneurysm of the vein of galen” OR “aneurysms of the vein of g	73378
#6	S4 OR S5	74227
#7	S3 OR S6	71
